# Strain rate dependency of dislocation plasticity

**DOI:** 10.1038/s41467-021-21939-1

**Published:** 2021-03-23

**Authors:** Haidong Fan, Qingyuan Wang, Jaafar A. El-Awady, Dierk Raabe, Michael Zaiser

**Affiliations:** 1 Department of Mechanics, Sichuan University, Chengdu, China; 2 Department Microstructure Physics and Alloy Design, Max-Planck-Institut für Eisenforschung GmbH, Düsseldorf, Germany; 3 Department of Mechanical Engineering, Whiting School of Engineering, The Johns Hopkins University, Baltimore, MD USA; 4 WW8-Materials Simulation, Department of Materials Science, FAU Universität Erlangen-Nürnberg, Fürth, Germany

**Keywords:** Molecular dynamics, Mechanical properties, Metals and alloys

## Abstract

Dislocation glide is a general deformation mode, governing the strength of metals. Via discrete dislocation dynamics and molecular dynamics simulations, we investigate the strain rate and dislocation density dependence of the strength of bulk copper and aluminum single crystals. An analytical relationship between material strength, dislocation density, strain rate and dislocation mobility is proposed, which agrees well with current simulations and published experiments. Results show that material strength displays a decreasing regime (strain rate hardening) and then increasing regime (classical forest hardening) as the dislocation density increases. Accordingly, the strength displays universally, as the strain rate increases, a strain rate-independent regime followed by a strain rate hardening regime. All results are captured by a single scaling function, which relates the scaled strength to a coupling parameter between dislocation density and strain rate. Such coupling parameter also controls the localization of plasticity, fluctuations of dislocation flow and distribution of dislocation velocity.

## Abstract (web summary)

The relationship between the strain rate and micro-scale deformation in metals is still poorly understood. Here the authors use discrete dislocation dynamics and molecular dynamics to establish a universal relationship between material strength, dislocation density, strain rate and dislocation mobility in fcc metals.

## Introduction

Metals are mostly used for their excellent load-bearing capacity, enabled by their mechanical strength and damage tolerance. Serving in practically all engineering fields such as transportation, energy, health, construction, and safety, they create an annual global market above 3000 billion Euros^1^. In many safety-relevant loading scenarios, the in-service mechanical response of metals depends significantly on loading rate, for instance, during vehicle crash, metal forming, medical implants or bird strike impact on jet engines. A strain rate hardening response is generic for metallic materials deforming by dislocation slip^2^, with exception of a limited regime of deformation conditions in solution-hardened alloys where dislocation-solute interactions may lead to strain rate softening^3,4^. Nevertheless, the relationship between the strain rate and microscale deformation mechanisms is still poorly understood, and most dynamic constitutive models (e.g., Johnson-Cook, Zerilli-Armstrong) were formulated in a phenomenological or semi-phenomenological manner with several empirical parameters that do not reflect microscale deformation mechanisms and need to be fitted to specific experiments with loss of generality^5^. Therefore, it is essential to develop a general understanding of the microscopic mechanisms that control strain rate effects, in order to develop physics-based models that are able to reflect and predict the strain rate dependence of the mechanical properties of metals. In BCC (body-centered cubic) metals, such as many steels, strain rate effects are often related to dislocation core properties (the relatively high atomic-scale Peierls barriers and the associated kink-pair mechanism), which control screw dislocation motion. The resulting temperature and stress dependent mobility of screw dislocations has been incorporated into numerous physics-based plasticity models (see refs. ^6,7^). In FCC (face-centered cubic) metals, such as Al and Cu, where dislocation motion is controlled by phonon drag, the situation becomes more complicated because dislocation motion is strongly affected by various collective phenomena related to the mutual elastic interactions among the dislocations. Investigating these phenomena and establishing their strain rate dependence is the aim of the present study.

Experimental studies on single-crystalline Cu^8^, Al^9^, and LiF^10^ as well as on polycrystalline Cu^11,12^, Al^5^ spanning many orders of magnitude in strain rate showed that the flow stress exhibits a weakly strain rate-dependent response at low strain rates followed by a strain rate hardening response at high strain rates. It has been argued that the strain rate-independent regime is dominated by dislocation forest interactions and/or dislocation interactions with grain boundaries or precipitates. On the other hand, the strain rate hardening regime was attributed to viscous drag forces acting on dislocations^5^. In this case, the stress acting on dislocations was related to the dislocation velocity through the dislocation drag coefficient, and the dislocation velocity to the strain rate through the Orowan relationship. Accordingly, the direct relationship between stress and strain rate depends on the ratio between the drag coefficient and the density of ‘mobile’ dislocations. This poses serious problems: drag coefficients predicted from strain rate-dependent stress–strain curves, under the assumption that all dislocations are mobile, are always significantly higher than theoretical estimates, and also higher than drag coefficients deduced from direct velocity measurements^13,14^. Such discrepancy persists even if additional scattering mechanisms beyond viscous phonon drag are considered^15–17^. Kumar et al. conversely used measurements of strain rate-dependent stress–strain curves in conjunction with directly measured drag coefficients to determine mobile dislocation densities, leading to a very low fraction of mobile dislocations, ~10^−5^ ^18^. The problem in all these studies resides in the fact that the mobile dislocation density is not a directly observable quantity. Also, it may be argued that the attribute ‘mobile’ is somewhat ill-defined since, depending on the loading conditions, any dislocation (including those were temporarily rendered immobile) can become mobile again. This is particularly important when load path or strain rate changes are imposed. As a consequence of the conceptual difficulties engendered by introducing the distinct categories of ‘mobile’ and ‘immobile’ dislocations, many fundamental questions regarding the relationship between the externally imposed strain rate and the internal collective dynamics of dislocations have never been properly answered. These questions concern not only the relationship between strain rate and average dislocation velocity and its dependence on dislocation density, but also the relationship between individual and collective dislocation behaviors. To settle these questions, a systematic investigation is required that focuses on the problem: how dislocations move.

Discrete dislocation dynamics (DDD) simulations allow in situ observations of collective dislocation behavior during plastic flow and can therefore provide fundamental insights into the mechanisms controlling strain rate effects of dislocation-mediated plasticity without the need of relying on ad hoc assumptions. In DDD simulations^19–23^, dislocations are coarse-grained as discrete elastic lines and most relevant dislocation mechanisms are accounted for in a physics-based fashion (dislocation glide, cross-slip, multiplication, annihilation, long-range interaction, junction formation, etc.). Over the past two decades, DDD has been extensively employed to investigate various aspects of dislocation-mediated plasticity. The two dimensional (2D) DDD approach was previously employed to study dislocation mobility at high strain rates^24^, and showed that dislocation inertia effects may be important for the accurate prediction of the dynamical properties of dislocations at high strain rates above 10^5^ s^−1^ ^25^. Using three-dimensional (3D) DDD simulations combined with finite element method, Liu et al. observed that dislocation patterns change from nonuniform to uniform under high strain rates^26^. Wang et al. performed 3D-DDD simulations and found that while almost all dislocations are mobile at high strain rates^27^, a very small percentage of the dislocations move at a speed approaching the shear wave velocity^28^. Under shock loading at super high strain rates, dislocation homogeneous nucleation plays an important role in dynamical plasticity^29,30^. 3D-DDD simulations were also employed to study shock deformation in silicon crystals under laser shock peening, and the dislocation density and dislocation multiplication rate are strongly dependent on the laser processing conditions^31^. While DDD simulations were applied to a wide range of problems in dislocation plasticity, the aforementioned fundamental questions pertaining to strain rate dependency have not been systematically investigated. Especially, essential quantities such as the mean dislocation velocity and distribution of dislocation velocity, which are difficult to be determined experimentally, were rarely studied, although they can be naturally obtained from 3D-DDD simulations.

In this work, we perform a total of 194 simulations using 3D-DDD and MD (molecular dynamics) methods to analyze the strain rate dependence of collective dislocation plasticity. In these simulations, the effects of dislocation density (varied over 9 orders of magnitude) and strain rate (varied over 10 orders of magnitude) on the plastic deformation behavior of bulk copper and aluminum single crystals are studied. The mean dislocation velocity and velocity distribution are analyzed in detail and universal characteristics of collective dislocation behavior are revealed. Based on this comprehensive database, we derive a universal analytical relationship between dislocation density, strain rate, material strength, and dislocation mobility, which predicts strain rate and dislocation density effects on the plastic properties of metals in terms of a single parameter that combines dislocation density and strain rate.

## Results

In this work, we performed a total of 194 DDD/MD simulations to study the material strength at different initial dislocation densities and strain rates. The initial dislocation density *ρ*_0_ was varied from 2.3 × 10^7^ m^−2^ to 2.2 × 10^16^ m^−2^ and strain rate $\dot \varepsilon$ from 0.1 s^−1^ to 2.5 × 10^8^ s^−1^. In the DDD/MD simulations, we simultaneously record stress, dislocation density, plastic strain, and mean dislocation velocity as functions of the loading strain $\dot \varepsilon t$. The corresponding datasets are provided in the Supplementary Data 1. For convenience of the readers, a compilation of all stress-stain curves is shown in Supplementary Fig. 4. Representative dislocation configurations are displayed in Supplementary Fig. 5 and Fig. 6.

### Material strength

We first analyze the dependency of the yield stress on dislocation density and strain rate. By default we define the axial yield stress *σ*_y_ as the axial stress at a plastic strain of $\varepsilon _y^p = 0.2\%$. Lower offset plastic strains of 0.001%, 0.01%, 0.05% are used for strain rates of 10^−1^s^−1^, 10^0^s^−1^, 10^1^s^−1^, respectively. The rationale for this procedure is discussed in Supplementary Note 1. The resolved shear stress at yield (short: yield stress) is *τ*_y_ = *mσ*_y_, where $m = 1/\sqrt 6$ is the Schmidt factor for the geometry used in our simulations. The dislocation density at yield is denoted as *ρ*_y_. It is worth noting that the dislocation density at yield is not exactly the same as the initial dislocation density *ρ*_0_ because the dislocation configuration changes both in the initial relaxation step and during loading. As a consequence, different initial configurations with the same initial density *ρ*_0_ lead to slight variations in yield density *ρ*_y_.

**Fig. 1:**
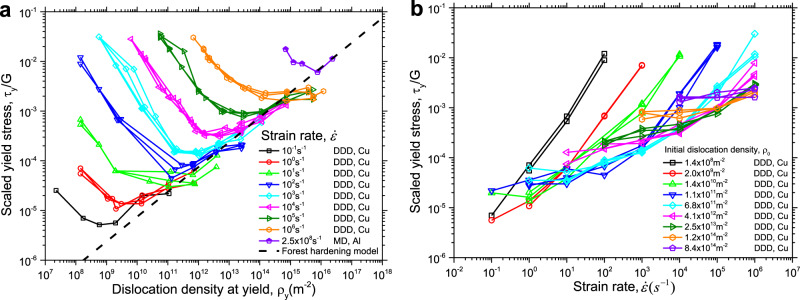
Yield stress as predicted from current DDD/MD simulations. **a** Scaled yield stress as a function of dislocation density at yield for different strain rates. **b** Scaled yield stress as a function of strain rate for different initial dislocation densities. In Fig. **b**, no MD points are shown because the MD simulations were conducted at only one strain rate. DDD is abbreviation of discrete dislocation dynamics and MD is molecular dynamics.

Figure 1 shows the yield stress *τ*_y_ as a function of dislocation density at yield, *ρ*_y_, and imposed strain rate, $\dot \varepsilon$, in a double-logarithmic manner. In Fig. 1a, it is clearly seen that for a given strain rate the yield stress displays two distinct regimes above and below a critical dislocation density, *ρ*_c_. When *ρ*_y_ < *ρ*_c_, the yield stress decreases with increasing dislocation density, while for *ρ*_y_ > *ρ*_c_, the yield stress increases with increasing density. It is also interesting to note that *ρ*_c_ increases with increasing strain rate. In addition, the curves for *ρ*_y_ > *ρ*_c_ for all simulated strain rates collapse onto a single line, which coincides with the classical forest hardening model ($\tau _y = \alpha Gb\sqrt {\rho _y}$, with $\alpha \approx 0.3$ for FCC metals), indicating a strain rate-independent response above this critical dislocation density and the dominance of forest hardening in this regime. On the other hand, while the slopes of the curves for *ρ*_y_ < *ρ*_c_ are almost equal, *τ*_y_ increases significantly with increasing strain rate for a given *ρ*_y_, suggesting that in this regime the material is prone to strain rate hardening. In the limit of infinitesimally low strain rate (quasi-static loading), only the second regime remains, indicating that the classical forest hardening mechanism is obtained without the consideration of strain rate effects. Clearly, a competition exists between strain rate hardening and forest hardening. As a result, the material strength is controlled jointly by an internal variable (dislocation density) and an external variable (strain rate). As demonstrated by the purple data points in Fig. 1a, these two regimes are equally observed in DDD and MD simulations, suggesting that the current predictions are not sensitive to the specific simulation method.

The yield stress as a function of strain rate is shown in Fig. 1b for different initial dislocation densities. At first glance, we see a quite complex picture: For the low initial dislocation density of *ρ*_0_ = 1.4 × 10^8^ m^−2^, *τ*_y_ increases linearly with increasing strain rate (the slope is unity in the double-logarithmic plot) over the simulated strain rate range. At intermediate initial dislocation densities, the stress level attained at a given strain rate in the linear regime progressively decreases as the initial dislocation density increases. At the same time, we observe a crossover from a linear strain rate dependence at high strain rates towards a low strain rate regime where the slope in the double-logarithmic plot decreases with decreasing strain rate. This crossover shifts to higher strain rates as initial dislocation density increases. In the low strain rate regime, the curves approach a horizontal asymptote (strain rate-independent yield stress) with an asymptotic stress level that increases with increasing initial dislocation density. At the highest initial dislocation density shown in Fig. 1b, viz. *ρ*_0_ = 8.5 × 10^14^ m^−2^, the yield stress is almost strain rate independent over the entire range of simulated strain rates. In fact, as we shall demonstrate below, the crossover from strain rate-independent behavior to a linear strain rate dependence of the yield stress is a generic feature of the competition between strain rate hardening and forest hardening. Such crossover also agrees well with extensive experimental observations^8–10,32^. That the crossover cannot be observed for the lowest and highest initial dislocation densities is a consequence of the limited range of attainable strain rates in the current simulations.

To analyze the behavior observed in our simulations, we note that the stress rate relates to the strain rate and plastic strain rate through the simple equation

1 $$\dot \sigma = E(\dot \varepsilon - \dot \varepsilon ^{\mathrm{p}})$$

**Fig. 2:**
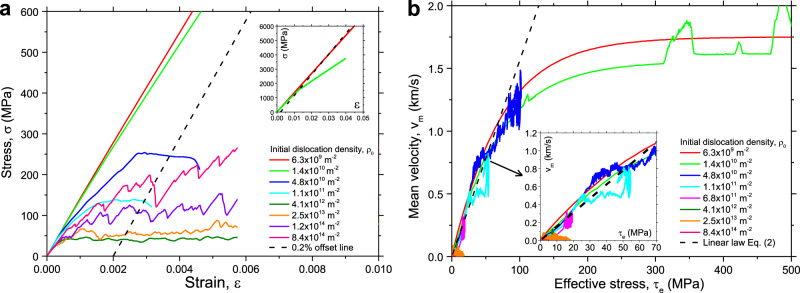
Stress–strain curves and mean dislocation velocity predicted from current DDD simulations. **a** Stress–strain curves at applied strain rate of 10^4^ s^−1^ and different initial dislocation densities. **b** Mean velocity of dislocations on active slip systems versus effective stress at applied strain rate of 10^4^ s^−1^ and different initial dislocation densities. DDD is abbreviation of discrete dislocation dynamics.

We first consider the behavior at extremely high strain rates and/or very low dislocation densities. Since the dislocation velocity cannot exceed the maximum value *v*_max_, for a given dislocation density *ρ*_y_, there exists an absolute upper limit of the plastic strain rate that can be accommodated by dislocation glide. This limit is given by $\dot \varepsilon _{\max }^p = m\rho _abv_{\max }$ (Orowan) where $\rho _a = f_a\rho _{\mathrm{y}} = 2\rho _{\mathrm{y}}/3$ is the dislocation density on the active slip systems. If a strain rate $\dot \varepsilon$ above this limit is imposed, Eq. (1) has no stationary solution and, hence, the stress is bound to increase indefinitely until, at an axial stress around 10 GPa, homogeneous dislocation nucleation sets in and the ensuing dramatic dislocation density increase allows to accommodate the imposed strain rate. We denote this scenario as the exhaustion regime of the strain rate dependent response, where the existing dislocations are insufficient to produce the imposed strain rate. Corresponding stress–strain curves are depicted in Fig. 2a, where the imposed strain rate lies above the plastic strain rate limit for the two lowest dislocation densities (red and green curves in the inset of Fig. 2a). As shown in the Supplementary Note 1, the yield stresses in this regime are proportional to the ratio $\dot \varepsilon /\rho _{\mathrm{y}}$.

Next, we move to lower strain rates or higher dislocation densities. Once the imposed strain rate falls below the plastic strain rate limit $\dot \varepsilon _{\max }^p = m\rho _abv_{\max }$, then Eq. (1) possesses a dislocation density dependent quasi-stationary solution, where the stress is implicitly related to the plastic strain rate via $\dot \varepsilon = \dot \varepsilon _{\,}^p = m\rho _abv_m(\tau )$ (*v*_m_ is the mean velocity of dislocations on the active slip systems). In Fig. 2a, all stress–strain curves with dislocation densities above 4.8 × 10^10^ m^−2^ fulfill this condition. These curves are characterized by a sharp transition between an elastic loading stage, and a plastic flow stage where the stress fluctuates around a nearly constant level.

Within the plastic flow regime, we again first look at the limit of low dislocation densities, where dislocation interactions can be neglected in comparison with the external stress needed to drive the dislocations against the lattice drag. This is referred to as the drag controlled regime. Since all dislocation velocities are well below the maximum velocity, the dislocation mobility law can be linearized, i.e., $v_m = (b/B)\tau$. Hence $\dot \varepsilon = f_am\tau \rho _{\mathrm{y}}b^2/B$ and $\tau = B\dot \varepsilon /(f_am\rho _{\mathrm{y}}b^2)$, which suggests again a linear relationship between the yield stress and the ratio $\dot \varepsilon /\rho _y$. We can see that both the exhaustion regime and the drag controlled regime possess the same dependence of stress on strain rate and dislocation density (for further discussion of this point see Supplementary Note 1), so they are henceforth jointly referred to as the strain rate hardening regime. The simulation data of Fig. 1a follow this behavior for low dislocation densities or high strain rates.

In the opposite limit of high dislocation density and/or low strain rate, the stress needed to drive dislocations is fully controlled by the mutual interactions of dislocations. In this forest hardening regime, the yield stress of any dislocation arrangement must follow the Taylor relationship, $\tau _y = \alpha Gb\sqrt {\rho _{\mathrm{y}}}$ (see ref. ^33^ for a general argument regarding this point). This relationship agrees well with the data in Fig. 1a in the regime of high dislocation densities and/or low strain rates.

The next question is whether the three different regimes can be unified into a consistent picture of the strain rate dependence of dislocation plasticity. A straightforward idea is that the mean driving stress for dislocation motion is given by an effective stress that equals the resolved shear stress, diminished by the dislocation resistance stress or Taylor stress: $\tau _{\mathrm{e}} = \tau - \alpha Gb\sqrt \rho$. We then expect that the mean dislocation velocity on the active slip systems follows the dislocation mobility law (Eq. (9)), with the local resolved shear stress replaced by the effective shear stress *τ*_e_. Figure 2b shows that the mean dislocation velocity follows well this prediction for a wide range of initial dislocation densities, as obtained from our DDD simulations. Outwith the exhaustion regime, the dislocation mobility law can be linearized, as shown in the inset of Fig. 2b. Accordingly,

2 $$v_m = \tau _eb/B = (\tau _y - \alpha Gb\sqrt {\rho _y} )b/B$$

Using Eqs. (1) and (2) and Orowan’s formula, it can be shown that

3A $$\tau _y = \frac{{B\dot \varepsilon }}{{mf_a\rho _yb^2}} + \alpha Gb\sqrt {\rho _y}$$

which can be alternatively expressed in terms of dimensionless variables to obtain a representation independent of material parameters:

3B $$T_1 = \frac{{\tau _y(mf_a)^{1/3}}}{{G^{2/3}(B\dot \varepsilon )^{1/3}}} = \frac{1}{{\rm{P}}} + \alpha \sqrt {\rm{P}} ,\,T_2 = \frac{{\tau _y}}{{Gb\sqrt {\rho _{\mathrm{y}}} }} = {\rm{E}} + \alpha$$

where the scaled dislocation density and strain rate are, respectively, given by

3C $${\rm{P}} = \left( {\frac{{mf_aGb^3}}{B}} \right)^{2/3}\frac{{\rho _{\mathrm{y}}}}{{\dot \varepsilon ^{2/3}}},\,{\rm{E}} = {\rm{P}}^{ - 3/2} = \frac{B}{{mf_aGb^3}}\frac{{\dot \varepsilon }}{{\rho _{\mathrm{y}}^{3/2}}}$$

**Fig. 3:**
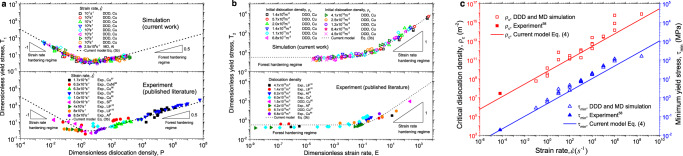
Comparison of our models, simulation data with published experiments^8–10,16,27,57–63^. **a** Dimensionless yield stress versus dimensionless dislocation density; **b** dimensionless yield stress versus dimensionless strain rate; **c** minimum yield stress and critical dislocation density at the transition point between forest hardening and strain rate hardening regimes, as a function of strain rate. All DDD/MD and experimental data presented in this figure are shown in Supplementary Data 2. DDD is abbreviation of discrete dislocation dynamics and MD is molecular dynamics.

Equation (3A) defines a dislocation kinetics model that provides a generic relationship between material strength, dislocation density, strain rate, and the related material parameters like dislocation mobility. This relationship can be stated in the universal forms of Eqs. (3B) and (3C) that are independent of material-specific parameters. As demonstrated in Fig. 3a-b, these unified models not only allow to collapse all the data in Fig. 1a, b onto two universal curves, but also help to aggregate data obtained for different materials both experimentally and by simulations into the same generic relationship. In particular, experimental data from different materials and for a wide range of deformation conditions follow the same generic curve as the DDD simulation data for Cu, and the same is true for MD simulation data obtained for Al. Technical aspects of the comparison with experiments are discussed in the Supplementary Note 3. In Supplementary Note 1, we also explain how, with the presently used yield definition based on a 0.2% plastic strain offset, Eq. (3) extend to the exhaustion regime.

In Eq. (3A-B), the second terms on the right-hand side control the mechanical behavior in the forest hardening regime at low strain rates (or high dislocation densities), and the first terms control the behavior in the strain rate hardening regime at high strain rates (or low dislocation densities). The transition between the two regimes can be identified with the minimum of the stress vs. dislocation density curve, which lies at ${\rm{P}} = {\rm{E}}^{ - 2/3} = (2/\alpha )^{2/3}$ in scaled representation. At this minimum, the forest hardening stress is exactly twice the strain rate hardening stress. The absolute values of the critical dislocation density and the minimum stress are given by

4 $$\rho _c = \left( {\frac{{2B\dot \varepsilon }}{{\alpha mf_aGb^3}}} \right)^{2/3}{\mathrm{and}}\,\tau _{{\mathrm{min}}} = \frac{3}{2}\alpha Gb\sqrt {\rho _c} = \left( {\frac{{27\alpha ^2G^2B\dot \varepsilon }}{{4mf_a}}} \right)^{1/3}$$

*τ*_min_ is the minimum material strength mediated by dislocation plasticity at a given strain rate, which is significant to the community of mechanics and materials. Figure 3c shows excellent agreement between the prediction of Eq. (4), the data from current DDD/MD simulations and published experimental results.

In many phenomenological plasticity models, the distinction between strain rate hardening and forest hardening terms is absent. Instead, the two-regime response is fitted over a limited range of strain rates by a power law relationship in the form of $\tau \propto \dot \varepsilon ^n$, where *n* is assumed to represent the strain rate sensitivity of the material. From our analysis it is clear that such a procedure does not adequately represent the intrinsic features of collective dislocation motion and is bound to produce misleading results. If, in Fig. 3b, one fits a linear relationship to the data over a limited range (say, two decades) of strain rates, then the linear approximation to the experimental and simulation data as well as to the theoretical curve (dashed line in Fig. 3b) may have any slope between 0 and 1 depending on the range of *E* values covered. Fits in the low-E regime are bound to produce low apparent strain rate sensitivities *n*, while fits in the regime of high E produce *n* values close to unity. Moreover, the *E* parameter depends systematically on dislocation density: lower dislocation densities correspond to higher values of *E* and thus are more likely to produce higher *n*. To establish the intrinsic material parameters that control the strain rate dependency of plastic flow, a different procedure is required: One needs first to subtract the forest hardening term (which is strain rate independent) from the measured stresses such as to produce a linear relationship between strain rate and stress. From this relationship one can determine the coefficient of the strain rate hardening term,

5 $${\mathrm{s}} = \frac{B}{{mf_a\rho b^2}}$$

which we propose as an adequate physical measure of strain rate sensitivity in plastic flow of FCC metals. From Eq. (5), the strain rate sensitivity is seen to be mainly controlled by the damping coefficient, *B*, and dislocation density, *ρ*, in a combination, which can explain many corresponding experimental observations in unified form. A higher dislocation density thus is expected to lead to lower strain rate sensitivity. This is in good agreement with experimental observations showing a decrease in strain rate sensitivity with increasing prestrain^10,34^. Also the strain rate sensitivity increases with increasing temperature for FCC crystals^34^ since the dislocation damping coefficient is linearly dependent on temperature^35^.

### Strain localization and patterning

**Fig. 4:**
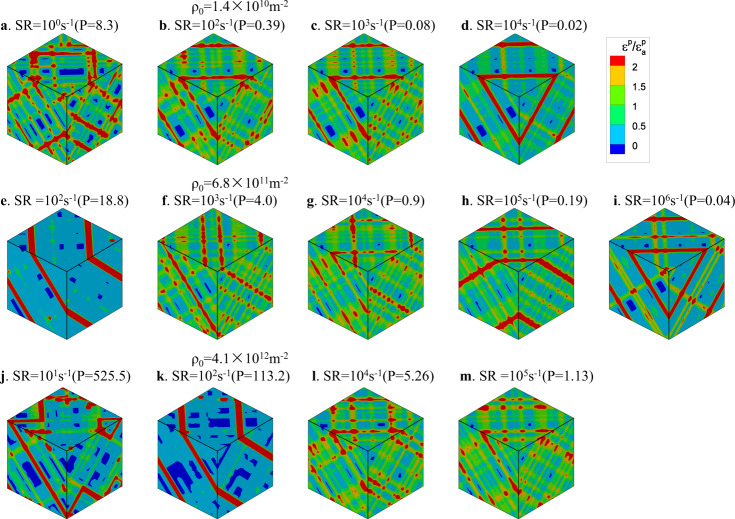
Contours of plastic strain for different initial dislocation densities and strain rates. In **a**. SR = 10^0^s^−1^, **b** SR = 10^2^s^−1^, **c** SR = 10^3^s^−1^ and **d** SR = 10^4^s^−1^, ρ_0_ = 1.4 × 10^10^m^−2^. In **e** SR = 10^2^s^−1^, **f** SR = 10^3^s^−1^, **g** SR = 10^4^s^−1^, **h** SR = 10^5^s^−1^ and **i** SR = 10^6^s^−1^, ρ_0_ = 6.8 × 10^11^m^−2^. In **j** SR = 10^1^s^−1^, **k** SR = 10^2^s^−1^, **l** SR = 10^4^s^−1^ and **m** SR = 10^5^s^−1^, ρ_0_ = 4.1 × 10^12^m^−2^. Plastic strain is given in units of mean plastic strain. In each row, the initial dislocation configuration is the same. The edge lengths of the depicted simulation boxes are 55.34 μm in the first row (**a**–**d**), 7.91 μm in the second row (**e**–**i**), 3.23 μm in the third row (**j**–**m**). SR is abbreviation of strain rate.

The dimensionless parameters, P and E, in Eq. (3C) not only govern the strain rate and dislocation density dependence of the yield stress, but also control the localization of plasticity. Contour plots of the local plastic strain are shown in Fig. 4 for different initial dislocation densities and strain rates. From Fig. 4, we can distinguish three regimes: plastic strain contrasts are strong at high dislocation density/low strain rate (high *P* > 10), also at low dislocation density/high strain rate (low *P* < 0.1). For 0.1 < *P* < 10, where the transition from the strain rate hardening regime to the forest hardening regime occurs, plastic strain is most homogeneous.

**Fig. 5:**
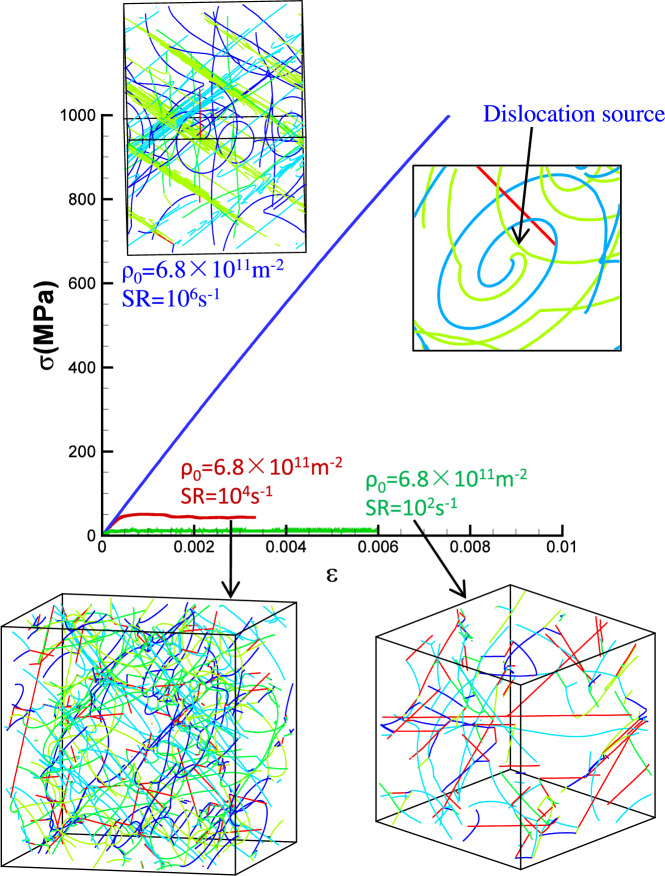
Dislocation configurations for same initial dislocation density but different strain rates. The shown simulation boxes have an edge length of 7.91 μm. The dislocation evolution processes in the three cases are shown in Supplementary Movies 1-3. SR is abbreviation of strain rate.

To explain the origin of the plastic strain patterns, the stress–strain curves and dislocation configurations for initial dislocation density of 6.8 × 10^11^ m^−2^ are shown in Fig. 5 for three typical strain rates. The simulation at the lowest strain rate of 10^2^ s^−1^ is in the forest hardening regime (Fig. 4e, *P* = 18.8, with pattern), the strain rate of 10^4^ s^−1^ is in the transition regime (Fig. 4g, *P* = 0.9, no pattern), while the strain rate of 10^6^ s^−1^ is in the exhaustion regime (Fig. 4i, *P* = 0.04, with pattern). The dislocation evolution processes in the three cases are shown in Supplementary Movies 1-3.

In Fig. 5, the stress–strain curve of 10^6^ s^−1^ is typical of the exhaustion regime. In this regime, existing dislocations are insufficient to produce the imposed strain rate, and the stress increases rapidly. Under the high stress, almost all dislocation junctions (red dislocations) formed during the initial relaxation are broken. Therefore, in the early yield stage, all dislocations move rapidly, as shown in Supplementary Movie 1, but their number is nevertheless insufficient to accommodate the imposed strain rate. The situation changes once dislocation sources are formed. As shown in Fig. 5, this happens by a collinear dislocation reaction, which leads to the formation of a single-armed source. At the high stress level that has been reached at that point, this process results in abundant dislocation multiplication on the source plane, as shown in Fig. 5 and movie 1. As a consequence, plastic strain localizes on the source plane (more generally: the source planes), leading to the observed, highly heterogeneous plastic strain patterns in Fig. 4i. One may thus conclude that, in the exhaustion regime, localization is driven by a multiplication instability which is sooner or later bound to happen because the stress increases dramatically, as the initially present dislocations are insufficient to accommodate the imposed strain rate. Once abundant dislocation multiplication sets in on a few slip planes, these dislocations dominate the deformation process, leading to the formation of pronounced slip bands as seen in Fig. 4i.

At the strain rate of 10^4^ s^−1^, which is at the transition between the two regimes of strain rate hardening and forest hardening, plastic flow is accommodated by continuous breaking and reformation of dislocation junctions, which lead to a quite homogeneous pattern of deformation. While the stress is high enough to ensure that most dislocations remain in motion, it is not high enough to enforce abundant dislocation multiplication and therefore deformation activity is approximately equal on all populated slip planes of the active slip systems. This is also seen from the near-homogeneous dislocation pattern shown in Fig. 5 for this strain rate and the corresponding Supplementary Movie 2.

The picture changes again when we go to even lower strain rates, i.e., 10^2^ s^−1^. Here the stress is just sufficient to break the weakest junctions, which are typically located in a region of reduced dislocation density. Deformation then proceeds mainly on the weakest slip planes, whose dislocations are sufficient to accommodate the low imposed strain rate (see Supplementary Movie 3). Again we observe localization (Fig. 4e), but it is driven by weakest-link behavior rather than dislocation multiplication. As dislocations move preferentially in dislocation depleted regions and get then entangled in dislocation dense regions, density fluctuations are enhanced. Further analysis of plastic strain heterogeneity in terms of the statistical distribution of the plastic strain is presented in Supplementary Note 4.

### Statistics of dislocation motion

**Fig. 6:**
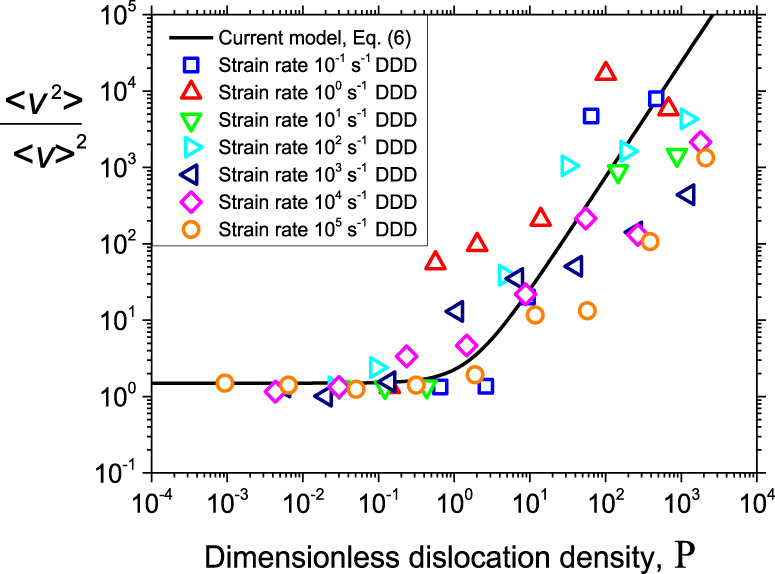
Squared variation coefficient of the dislocation velocity distribution in DDD simulations. Symbols are datasets for different strain rates and full line is the theoretical prediction of Eq. (6). DDD is abbreviation of discrete dislocation dynamics.

The dimensionless parameters, P and E, also control the statistics of dislocation motion. Again, we observe a clear distinction between forest hardening and strain rate hardening regimes. This is seen in Fig. 6, which shows the second moment $\left\langle {v^2} \right\rangle$ of the dislocation velocity distribution obtained from current DDD simulations, normalized by the square of the mean velocity $\left\langle v \right\rangle$ of all dislocations. In the strain rate hardening regime at low P values (*P* < 1), the mean square velocity is of the order of the mean velocity squared, i.e., fluctuations are small in absolute terms and the second moment of the velocity distribution is approximately independent on dislocation density. At high values (*P* » 1), the second moment of the velocity distribution grows as P^3/2^. A theoretical expression describing this behavior can be derived by analysing the microscopic energy dissipation (the work expended in moving dislocations against the drag force) and equating this to the macroscopic dissipated energy (the work expended macroscopically to create a plastic strain), as shown in the Supplementary Note 2, and the result reads in scaled notation.

6 $$\frac{{\left\langle {v^2} \right\rangle }}{{\left\langle v \right\rangle ^2}} = \frac{1}{{f_a}}\left( {\alpha {\rm{P}}^{3/2} + 1} \right) = \frac{1}{{f_a}}\left( {\frac{\alpha }{{\rm{E}}} + 1} \right)$$

As shown in Fig. 6, this relationship gives a good description of the increase of fluctuations in the regime of high dislocation densities and/or low strain rates that we observe in DDD simulations. Note that the left-hand side can be interpreted as a dissipation ratio, where the numerator is proportional to the actual dissipated energy, and the denominator is proportional to the fictitious dissipation in a hypothetical system of noninteracting dislocations of the same dislocation density and strain rate.

**Fig. 7:**
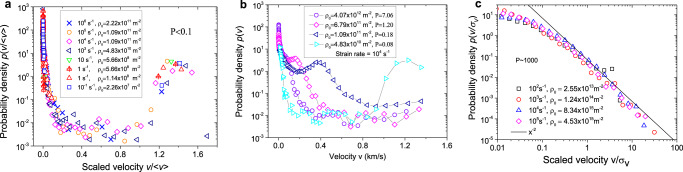
Probability distributions of dislocation velocities as observed in DDD simulations. **a** Distributions in the strain rate hardening regime, *P* < 0.1. **b** Distributions in the intermediate regime 0.1 < *P* < 10. **c** Distributions in the forest hardening regime, P ~ 1000, in double-logarithmic representation, where the full line indicates a slope of −2. *σ*_*v*_ is the standard deviation of dislocation velocity distribution, and $\left\langle v \right\rangle$is the mean velocity of all dislocations. DDD is abbreviation of discrete dislocation dynamics.

The dimensionless parameters P and E not only control the magnitude of fluctuations but also govern the statistics of the dislocation velocities: dislocation velocity distributions pertaining to the same P/E values are identical if properly rescaled. This is illustrated in Fig. 7 showing dislocation velocity distributions from current DDD simulations. In the strain rate hardening regime (Fig. 7a for a small value of P), we find bimodal distributions with one peak at near zero velocity which represents dislocations on inactive slip systems, and one peak at high velocity comprising all dislocations on active slip systems. For the second peak, the velocities of these dislocations are fairly uniform and scatter around the peak velocity value that is required to produce the imposed strain rate. From Eqs. (2) and (3A)

7 $$v_{{\mathrm{peak2}}} \approx v_{\mathrm{m}} = \frac{{\left\langle v \right\rangle }}{{f_a}}{\mathrm{,so}}\,\frac{{v_{{\mathrm{peak2}}}}}{{\left\langle v \right\rangle }} \approx \frac{1}{{f_a}}$$

The unified picture that emerges in the strain rate hardening regime is thus clear: we can distinguish immobile dislocations, which are the dislocations on the inactive slip systems, from mobile dislocations, which comprise all dislocations on the active slip systems. These dislocations move at the velocity needed to produce the imposed strain rate, with only minor velocity fluctuations. The stress response is dictated by the drag on dislocations, and dislocation–dislocation interactions are fairly unimportant.

As the dimensionless strain rate parameter E decreases or the density parameter P increases towards unity, the high-velocity peak of the velocity distribution shifts to lower values and ultimately merges, for high P, with the low-velocity peak (see Fig. 7b), leading to unimodal dislocation velocity distribution that is typical and characteristic of the forest hardening regime. In the strain rate-independent limit ${\rm{P}} \to \infty$ (see Fig. 7c), the velocity distribution acquires scale free features as the probability density *p*(*v*) decreases, for high velocity, in inverse proportion with *v*^2^, leading to the diverging fluctuations shown in Fig. 6.

## Discussion

In this work, the strain rate dependence of collective dislocation dynamics was studied using a large set of 3D-DDD (discrete dislocation dynamics) and MD (molecular dynamics) simulations, spanning nine orders of magnitude in initial dislocation density and ten orders of magnitude in strain rate. The performed 194 simulations indicate that the material strength displays two regimes, a strain rate hardening regime where the yield stress increases in proportion with strain rate and in inverse proportion with dislocation density at yield, and a regime of classical forest hardening where the yield stress is approximately strain rate independent and follows the Taylor relationship. All results can be described in terms of a scaled dislocation density P $(\rho _y/\dot \varepsilon ^{2/3})$ or strain rate E (${\rm{P}}^{ - 3/2}$), which combine dislocation density and strain rate, in such a manner that the corresponding yield stress can be expressed through a universal material independent relationship. The analytical relationship describes not only our simulations over the entire range of strain rates and dislocation densities, but also a wide range of experimental data published in the literature. The dimensionless parameters, P and E, also control the localization of plasticity. Plastic strain is localized at high dislocation density/low strain rate (high *P* > 10, forest hardening regime), and at low dislocation density/high strain rate (low *P* < 0.1, strain rate hardening regime). For 0.1 < *P* < 10, where the transition of both regimes occurs, plastic strain is most homogeneous. The dimensionless parameters, P and E, also govern the statistics of dislocation velocities. In the strain rate hardening regime of high strain rates/low dislocation densities, we find bimodal velocity distributions, where dislocations on inactive slip systems remain immobile whereas dislocations on active slip systems move with small fluctuations in a quasi-laminar manner at the velocity needed to match the imposed strain rate. In the forest hardening regime, on the other hand, the velocity distributions have scale free characteristics and decrease monotonically towards high velocity according to a $p(v) \propto v^{ - 2}$ power law.

The current results have far-reaching consequences both regarding the interpretation of experiments and the constitutive modeling of crystal plasticity. The interpretation of experiments that try to probe the strain rate dependence of dislocation motion and to establish drag coefficients has hinged on the idea that it is possible to distinguish a mobile dislocation density, which moves at a velocity that is dictated by the externally applied stress, and an immobile dislocation density that consists of dislocations remaining essentially stationary. Our investigation demonstrates that such a distinction makes sense only in the strain rate hardening regime of high strain rates and/or low dislocation densities. Only experiments conducted in this regime can yield results that are amenable to direct interpretation. However, most actual experiments have been conducted at low strain rates and/or high dislocation densities, i.e., in the forest hardening regime (see Fig. 3a). In these cases, identifying the mobile dislocation density is bound to systematically overestimate drag effects, and the introduction of a mobile fraction of the dislocation density is tantamount to introducing a variable that cannot easily be determined independently either by experiments or, as our study demonstrates, in simulations. At the same time, our results offer a way out of this dilemma, as we provide a universal yield stress relationship, which contains only the total dislocation density and strain rate (both measurable quantities) together with material parameters. One of these parameters is the poorly documented drag coefficient (as discussed in the introduction), whereas the remaining parameters (shear modulus, Burgers vector) are accurately known. Thus, by rescaling experimental data obtained from samples at different strain rates to fall on the master curve provided by our Eq. (3) and depicted in Fig. 3, it is possible to determine the drag coefficient *B* without having to assume a value for the mobile dislocation density.

Regarding constitutive modeling, we note that, starting from the Kubin-Estrin model^36^, many dislocation based crystal plasticity models contain a ‘mobile dislocation density’ as a constitutive variable (for recent examples, see ref. ^37^). Our analysis demonstrates that, in the forest hardening regime, the distribution of dislocation segment velocities alone, which is scale free and not bimodal, offers no obvious means to define such a quantity, even though definitions based on dislocation core properties or simply by appropriate thresholding of the velocity might be possible. In the strain rate hardening regime, by contrast, the mobile dislocation density simply encompasses almost all dislocations on active slip systems.

We finally note that the transition between the forest hardening and strain rate hardening regimes of dislocation plasticity not only affects the manner in which the flow stress depends on dislocation density and strain rate, and the magnitude of dislocation velocity fluctuations, but has important consequences regarding the formation of spatially inhomogeneous strain and dislocation patterns, which occur both in the exhaustion regime and the forest hardening regime, but not in the intermediate regime. As noted by several authors^38,39^, homogeneous flow of dislocations is unstable with respect to formation of localized strain and dislocation density patterns in the forest hardening regime of low strain rates and/or high dislocation densities. In this regime the strain rate is a decreasing function of dislocation density, which is equivalent to the flow stress at constant strain rate being an increasing function of dislocation density. Wu and Zaiser (2020^40^) show explicitly that the critical condition for the formation of heterogeneous dislocation patterns during deformation in symmetrical double slip is identical with the condition for the transition from the strain rate hardening regime to the forest hardening regime established in Eq. (3) of the present work. At the same time, in the exhaustion regime of very low dislocation densities and high strain rates, deformation is characterized by a multiplication instability where dislocation exhaustion leads to very high stresses, which ultimately trigger abundant dislocation multiplication and strain localization on planes where dislocation sources are formed.

In summary, our investigation provides a unifying picture of the strain rate and dislocation density dependence of collective dislocation dynamics over a so far unprecedented range of scales. In the regime of comparatively low strain rates or high dislocation densities, in which most laboratory experiments are conducted, collective dynamics of dislocations appears as a highly turbulent flow process. Once a sufficiently high applied stress causes the dislocation arrangement to lose metastability, complex relaxation processes lead to highly irregular dynamics with a scale free dislocation velocity spectrum and a strong propensity to the formation of heterogeneous strain and dislocation patterns.

## Methods

3D-DDD simulations in this work were performed using the open source code, ParaDiS (v2.5.1), developed at Lawrence Livermore National Laboratory^20^. In ParaDiS, dislocations are discretized into sequences of individual interconnected dislocation segments, each of which carries elastic distortion and associated stress field. Under external applied load ***σ***_ex_, each dislocation segment experiences a force per unit length

8 $${\boldsymbol{F}} = {\boldsymbol{b}} \cdot \left( {{\mathbf{\sigma }}_{{\mathrm{ex}}} + {\mathbf{\sigma }}_{{\mathrm{dis}}}} \right) \times {\mathbf{\xi }} + {\boldsymbol{F}}_0 + {\boldsymbol{F}}_{{\mathrm{self}}}$$

where ***ξ*** is the dislocation line direction, ***b*** is the Burgers vector of the dislocation segment, ***σ***_dis_ is the long-range interaction stress between the current dislocation and others, ***F***_self_ is the dislocation self-force, and ***F***_0_ is the lattice friction force. Under this total force, each dislocation segment glides on its slip plane. During dislocation glide, short-range dislocation interactions are taken into account, including junction formation and breaking, cross-slip, dislocation annihilation and multiplication. The total plastic strain inside the simulation cell is calculated from the area *A* swept by the dislocation segments, $\varepsilon ^{\mathrm{p}} = {\sum} {\frac{A}{{2V}}} ({\mathbf{n}} \otimes {\mathbf{b}} + {\mathbf{b}} \otimes {\mathbf{n}})$, where **n** is the unit normal to the slip plane, *V* is the volume of the simulation cell. Then the response in stress can be obtained by $\sigma _{{\mathrm{ex}}} = E(\varepsilon - \varepsilon ^{\mathrm{p}})$. In recent years, ParaDiS was employed frequently to model crystal plasticity in various situations, such as bulk strain hardening, grain boundary strengthening, precipitation hardening and deformation twinning^41–44^.

Here, ParaDiS is used to quantify the strain rate effects on collective dislocation behavior in plastically deforming bulk copper (Cu) single crystals. All DDD simulations were conducted for cubic cells with periodic boundary conditions in three directions. The cube edges were aligned with the three orthogonal crystal directions *X* = [100], *Y* = [010], and *Z* = [001], respectively. To ensure bulk-like behavior and minimize artifacts induced by the periodic boundary conditions, the simulation cell size *L* must be several times larger than the characteristic wavelength of the microstructure (here the dislocation spacing which is estimated as the inverse square root of the initial dislocation density *ρ*_0_)^45^. Accordingly, the simulation cell size is adjusted to keep the ratio of simulation cell size to initial dislocation spacing $L\rho _0^{1/2} \,> \,4$, which leads to physical sizes, depending on *ρ*_0_, ranging from 1 mm for the lowest dislocation density of 2.3 × 10^7^ m^−2^ to 100 nm for the highest dislocation density of 1.4 × 10^16^ m^−2^. Increasing this ratio has no significant effects on the results as discussed in the Supplementary Note 1. The material parameters used in all DDD simulations are those of FCC Cu: shear modulus, *G* = 54.6 GPa; Poisson ratio, *υ* = 0.324; magnitude of Burgers vector, *b* = 0.25 nm.

**Fig. 8:**
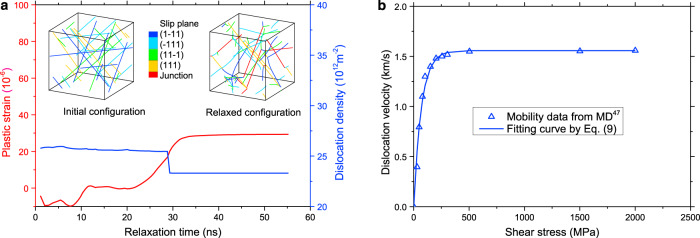
Plastic strain during relaxation and dislocation mobility law in 3D-DDD simulations. **a** Plastic strain during relaxation for a simulation with an initial dislocation density of *ρ*_0_ = 2.5 × 10^13^ m^−2^ in bulk copper. Insets show the initial dislocation configuration and relaxed configuration with dislocation junctions formed by reactions among the initially present dislocations. **b** Dislocation velocity versus resolved shear stress for an edge dislocation as predicted from MD simulations^47^, and the exponential dislocation mobility law shown in Eq. (9). Screw dislocation mobility is assumed equal to edge dislocation mobility. DDD is abbreviation of discrete dislocation dynamics and MD is molecular dynamics.

In many previous DDD simulations, the initial dislocation configurations consisted of Frank-Read dislocation sources (a dislocation ending at two pinning nodes)^46^. Such initial conditions are not only inconsistent with Burgers vector conservation, since the dislocation ends within the crystal, but might also cause artifacts in the dynamics, as the artificially introduced pinning nodes are much stronger than naturally formed ones. Therefore, here we introduced infinite-length dislocations spanning two periodic cells, which are equi-distributed over the 12 possible slip systems. A typical example of initial configuration is shown in Fig. 8a. The initial dislocation density, *ρ*_0_, was varied over nine orders of magnitude (2.3 × 10^7^ m^−2^∼1.4 × 10^16^ m^−2^). The initial dislocation configuration was first relaxed under zero stress until the incremental plastic strain is less than 10^−7^ in 10 *ns*. During the relaxation, the dislocation density decreases due to dislocation reactions driven by dislocation-related internal stresses. Figure 8a shows that the plastic strain is approaching saturation, indicating that the dislocation configuration approaches a stable state. A representative relaxed dislocation network shown in the inset of Fig. 8a exhibits a large number of naturally forming dislocation junctions with a very wide spectrum of junction lengths. It should be noted that the accumulated plastic strain produced during the relaxation is significant (up to 0.12% in simulations with a high initial dislocation density). If the relaxation would be omitted, this accumulative plastic strain would show as a prestrain occurring during the elastic loading stage. Thus, the initial relaxation is important to accurately represent a crystal in equilibrium. Then, a constant strain rate $\dot \varepsilon$ is imposed parallel to the simulation cell edge along the *Z* direction. The imposed strain rate was varied by 7 orders of magnitude from 0.1 s^−1^ to 10^6^ s^−1^. To account for the effect of variations in the initial dislocation network, each simulation was run at least three times (except the case of 0.1 s^−1^) with the same initial dislocation density but different random distributions of the initial dislocations. A total of 189 simulations were thus performed.

In high strain rate experiments, the stress is believed to be closely related to the mobility of dislocations^13,14^. Thus, to accurately predict dislocation kinetics in high strain rate simulations, a realistic dislocation mobility law is needed. Recent MD simulations of edge dislocation velocity versus resolved shear stress in Cu^47^, reproduced in Fig. 8b, show a nonlinear dislocation mobility relationship. Screw dislocation mobility is comparable with edge dislocation mobility. In the current DDD simulations, we utilize an exponential mobility rule of the form^48^

9 $$v = v_{{\mathrm{max}}}(1 - {\mathrm{exp}}( - k\tau ))$$

where *v*_max_ = 1.5579 km/s is the upper limit of the dislocation velocity and *k* = 0.0146/MPa is a constant. This mobility law matches reasonably well the MD predictions (see Fig. 8b). It is worth noting that the functional form of Eq. (9) also provides a good fit for velocity-stress curves in other FCC metals (e.g., Ni, Al, and Al/Mg alloys)^35^. We finally note that this mobility law reduces, in the regime of low to intermediate velocity, to the often-used linear drag law, *v* = *bτ*/*B*, with linear drag coefficient *B* = 1.6 × 10^−5^ Pa s. At the same time, the exponential saturation avoids unphysical behavior that would otherwise occurs associated with dislocations passing the sound velocity barrier. For validation purposes, we compare our results with MD simulations, where effects of dislocation inertia and relativistic effects^49^ (i.e., the effective mass of a dislocation diverges as the dislocation approaches the sound velocity) are naturally included. This comparison demonstrates that Eq. (9) provides an adequate representation of collective dislocation behavior even in the high-velocity regime.

To ensure that the current predictions are not contingent on simulation method, large scale MD simulations were conducted using the MD simulation package LAMMPS^50^, with the atomic potential for FCC Al^51^. The cubic simulation cell has a size of 113.4 nm with periodic boundary conditions applied in three directions and contains 88 million atoms. In the MD simulation cell, we initially introduced dislocation loops with the same size as the simulation cell^52^. Five initial dislocation densities were considered from 3.5 × 10^14^ m^−2^ to 2.2 × 10^16^ m^−2^. After a relaxation achieved through a conjugate gradient algorithm, a dislocation network forms. Then a strain rate of 2.5 × 10^8^ s^−1^ was applied on the simulation cell to study the dislocation dynamics during plastic flow.

In all DDD and MD simulations, only dislocation-mediated plasticity has been considered since other deformation modes (e.g., twinning) are active at shock loading stresses in excess of 35 GPa^53,54^ or strain rate in excess of 1.25 × 10^9^ s^−1^ ^52^, a regime that is beyond the stresses and strain rates of interest in this study. For the same reason, homogenous dislocation nucleation in the crystal was also neglected since previous MD simulations^55^ indicate a homogenous nucleation stress of ~10 GPa in copper, which is higher than all the yield stresses reached in the current simulations. Finally, we note that the specimen acceleration effect is less than 2% of the dynamic stress for strain rate below 10^5^ s^−1^ ^56^, and was not considered in the DDD simulations. Dislocation cross-slip is not included, which is important at high strain levels.

## Supplementary information

Supplementary Information

Description of Additional Supplementary Files

Supplementary Data 1

Supplementary Data 2

Supplementary Movie 1

Supplementary Movie 2

Supplementary Movie 3

## Data Availability

All the other raw data required to reproduce these findings are available from authors upon request. The ParaDiS v2.5.1 was downloaded from http://paradis.stanford.edu.
