# Author Correction: Strain rate dependency of dislocation plasticity

**DOI:** 10.1038/s41467-021-22963-x

**Published:** 2021-04-23

**Authors:** Haidong Fan, Qingyuan Wang, Jaafar A. El-Awady, Dierk Raabe, Michael Zaiser

**Affiliations:** 1 Department of Mechanics, Sichuan University, Chengdu, China; 2 Department Microstructure Physics and Alloy Design, Max-Planck-Institut für Eisenforschung GmbH, Düsseldorf, Germany; 3 Department of Mechanical Engineering, Whiting School of Engineering, The Johns Hopkins University, Baltimore, MD USA; 4 WW8-Materials Simulation, Department of Materials Science, FAU Universität Erlangen-Nürnberg, Fürth, Germany

**Keywords:** Molecular dynamics, Mechanical properties, Metals and alloys

Correction to: *Nature Communications* 10.1038/s41467-021-21939-1, published online 23 March 2021

The original version of this Article contained an error in Figs. 3 and 8.

In the original version of Fig. 3, the references reported in panels a and c are not correct.

The correct version of Fig. 3 is:
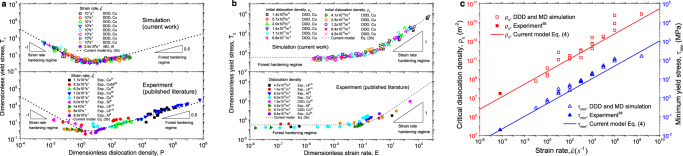


which replaces the previous incorrect version:
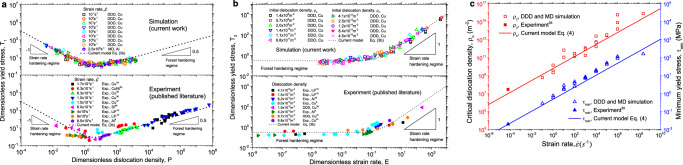


In the original version of Fig. 8, the reference reported in panel b is not correct.

The correct version of Fig. 8 is:
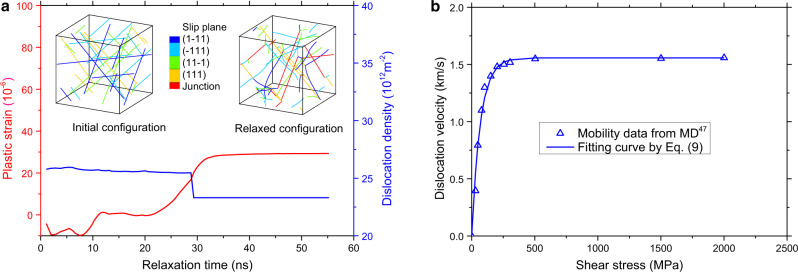


which replaces the previous incorrect version:
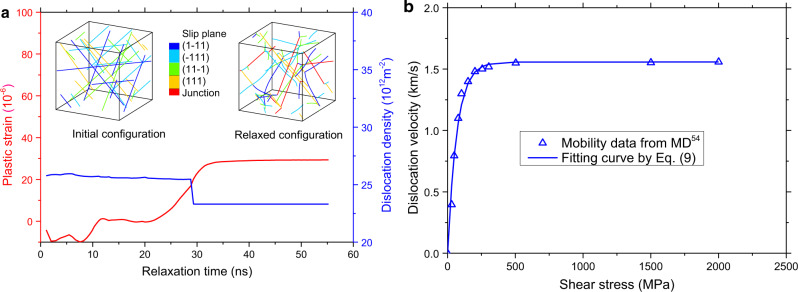


This has been corrected in both the PDF and HTML versions of the Article.

