# Supplementary material for: Strain rate dependency of dislocation plasticity
Source: Nat Commun. 2021 Mar 23;12:1845. doi: 10.1038/s41467-021-21939-1 (PMC7988163; doi:10.1038/s41467-021-21939-1)
Supplement: Supplementary file 1 — Supplementary Information [file 41467_2021_21939_MOESM1_ESM.pdf]

Strain rate dependency of dislocation plasticity

Haidong Fan[1,2*], Qingyuan Wang[1*], Jaafar A. El-Awady[3], Dierk Raabe[2], Michael Zaiser[4]

[1] Department of Mechanics, Sichuan University, Chengdu 610065, China

[2] Department Microstructure Physics and Alloy Design, Max-Planck-Institut für Eisenforschung GmbH, Düsseldorf 40237, Germany

[3] Department of Mechanical Engineering, Whiting School of Engineering, The Johns Hopkins University, Baltimore, MD 21218, USA

[4] WW8-Materials Simulation, Department of Materials Science, FAU Universität Erlangen-Nürnberg, Fürth 90762, Germany

*Corresponding authors emails: hfan85@scu.edu.cn (HF), wangqy@scu.edu.cn (QW)

Supplementary Note 1: Simulation method

Since we conducted serial simulations over a very wide range of dislocation densities and strain rates, it is mandatory to keep the computational cost per simulation as low as possible. This indicates to use a small simulation cell size L, which, however, must be at the same time large enough for the dislocations to sample configurations representative of bulk behavior both during initial relaxation and during loading. Because of scaling relations governing dislocation simulations[1], simulation cell size must, in this context, be measured in units of dislocation spacing $\rho_0^{-1/2}$, i.e., in terms of the ratio $r = L\rho_0^{1/2}$ where ρ_0 is the initial dislocation density. To assess acceptable values of r, we systematically varied this parameter as shown in Supplementary Fig. 1. We conclude that, while there is a very significant r dependence of the stress-strain curves for very small r, for values $r \geq 4$ there is no systematic change of the stress-strain curves if the simulation cell size is further increased. This leads us to choose r=4 as default cell size in our simulations.

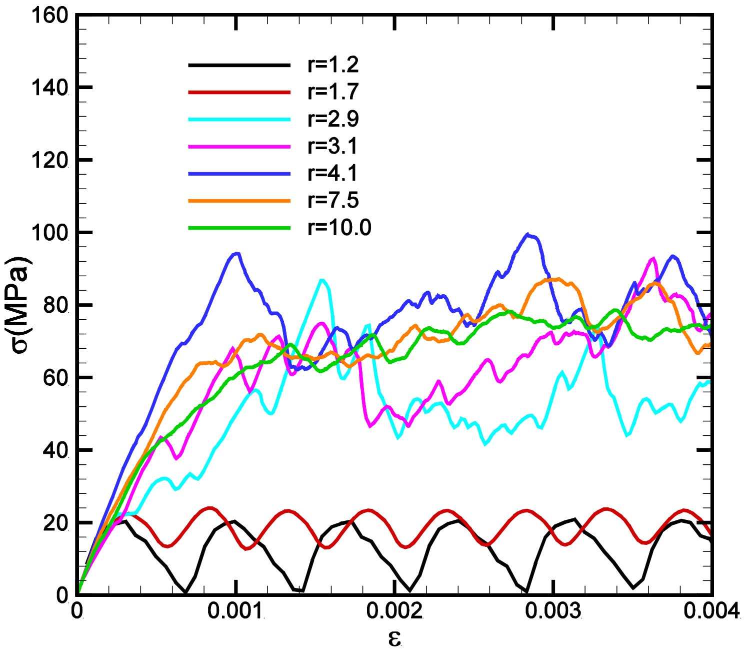

Supplementary Figure 1. Stress-strain curves at different ratios of simulation cell size over dislocation spacing. $\dot{\varepsilon}=10^4 s^{-1}$, $\rho_0=2.3\times10^{13} m^{-2}$.

In engineering, the yield stress is defined as the stress at the point on the stress-strain

curve where a prescribed plastic strain value (e.g., 0.2% plastic strain) is reached. While this offset is normally chosen by convention, we aim to make sure that it correctly reflects basic aspects of dislocation behavior. To this end we consider the two extreme regimes (forest hardening regime vs exhaustion regime) separately.

In the forest hardening regime, yielding can, in dislocation terms, be defined as the stress driven transition of the dislocation system from an initial, stable configuration of the dislocation network (energy minimum) to a flowing state. For an initially random dislocation system as considered in our simulations, the mean glide distance of dislocations between two statistically independent energy minima simply equals the mean dislocation spacing $\rho_0^{-1/2}$. Accordingly, the saddle point, which marks the transition out of the original minimum energy configuration, is reached, if all dislocations on the active slip systems move by half a dislocation spacing on average, $\Delta l = 1/(2\rho_0^{1/2})$. The corresponding axial strain serves as an estimated lower bound to the plastic strain that should be accomplished at yield:

$$\varepsilon^p \geq \varepsilon_{\mathrm{c}}^{\mathrm{p}} = \frac{m}{3} b \sqrt{\rho_0} \tag{1}$$

If plastic strain is below this level, dislocations may, upon unloading, relax back into their initial configurations, fully or partly reversing the dislocation related plastic strain. On the other hand, higher plastic strain offsets are acceptable. They merely ensure that the dislocation system explores a larger number of configurations, which leads to oscillations of the instantaneous stress level around a near-constant value.

A different situation is encountered in the exhaustion regime, where dislocation interactions are almost irrelevant as high stresses drive the dislocations at high velocities approaching the maximum velocity (i.e. $v_m \approx v_{\max}$). The stress rate is given by $\dot{\sigma} = E(\dot{\varepsilon} - \dot{\varepsilon}^{\mathrm{p}})$. Dividing by $\dot{\varepsilon}$ and using Orowan's formula, the slope $\theta = d\sigma / d\varepsilon$ of the stress-strain curve follows as

$$\theta = E(1 - m f_a \rho_y b v_{\max}/\dot{\varepsilon}) \tag{2}$$

On the other hand, the axial yield stress is related to the plastic strain offset $\varepsilon_{\mathrm{y}}^{\mathrm{p}}$ by $\sigma_{\mathrm{y}} = E(\varepsilon - \varepsilon_{\mathrm{y}}^{\mathrm{p}})$ where $\varepsilon = \sigma_{\mathrm{y}} / \theta$. Eliminating θ results in

$$\tau_y = m\sigma_y = mE\varepsilon_y^p\left[\frac{\dot{\varepsilon}}{mf_a\rho_y b v_{max}} - 1\right] \quad (3)$$

To obtain an appropriate definition of the yield plastic strain ε_y^p, we note that according to Supplementary Eq. (3) the yield stress in the exhaustion regime is linearly proportional to $\dot{\varepsilon}/\rho_y$, which is similar to the behavior in the drag controlled regime, where $\tau_y = B\dot{\varepsilon}/(f_a m\rho_y b^2)$ (see Eq. (3a) of the main paper and note the forest hardening stress $\alpha Gb\sqrt{\rho_y}$ is insignificant here). Both expressions can be matched by setting $\varepsilon_y^p = Bv_{max}/(mbE)$. As shown in the theory of dislocations[2], the damping coefficient can be expressed as $B = \eta Gb/v_{max}$, where $\eta \approx 0.002$. This ultimately leads to the result that an appropriate choice, which unifies the behavior in the drag controlled and exhaustion regimes, is given by

$$\varepsilon_y^p = \frac{\eta}{2m(1+\upsilon)} \approx 0.002 \quad . \quad (4)$$

Coming back to the forest hardening regime, upon inserting this value into Supplementary Eq. (1), we see that the inequality $\varepsilon_y^p = 0.002 \geq \varepsilon_c^p$ is fulfilled for dislocation densities up to $\rho_0 \approx 10^{16}\,\mathrm{m}^{-2}$, i.e., for the entire range of initial dislocation densities covered in our study. This leads us to use $\varepsilon_y^p = 0.2\%$ as the default yield plastic strain in our study.

At very low strain rates, however, a practical problem arises, which stems from the computational cost of DDD simulations again. As is well-known, the total computational cost of DDD simulations increases significantly as one moves to low strain rates. This makes it very challenging to conduct dislocation simulations at strain rates as low as $0.1\mathrm{s}^{-1}$. Such a low strain rate is easy to run in a simulation with only one dislocation[3], but has been very rarely reported in the literature for simulations of collective dislocation behavior. To mitigate the problem of excessive simulation time, at the lowest strain rates of 0.1 s^{-1}, 1 s^{-1} and 10 s^{-1}, we use reduced yield plastic strains of ε_y^p=0.001%, 0.01% and 0.05%, respectively. These plastic strains all fulfill, for the dislocation densities simulated at the respective strain rates, the

condition given by Supplementary Eq. (1). Accordingly, as illustrated in Supplementary Fig. 2, the stress-strain curves are, at these plastic strain levels, near-horizontal. Thus, an increase of ε_y^p would not change the results, which are well described by the current dislocation kinetics model (dashed lines in Supplementary Fig. 2, Eq. (3a) of the main paper), which indicates again that the choice of lower plastic strains is reasonable.

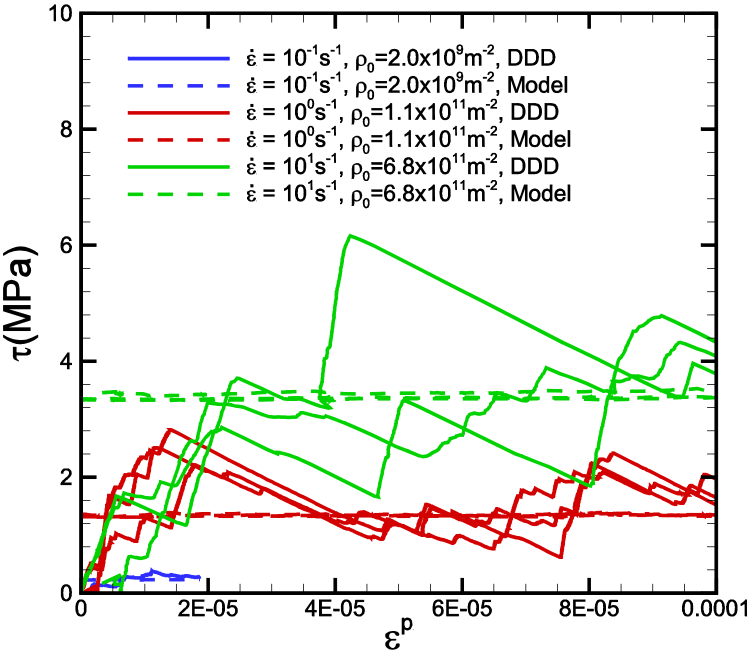

Supplementary Figure 2. Shear stresses from DDD simulations and model (Eq. (3a) of the main paper). The model curves reflect predictions of the model evaluated from the plastic strain dependent instantaneous dislocation density, hence there are multiple curves for each strain rate. DDD is abbreviation of discrete dislocation dynamics.

Supplementary Note 2: A fluctuation-dissipation theorem for dislocation plasticity

It is a standard assumption in continuum plasticity theory, which is motivated by thermodynamic arguments, that the work expended in creating plastic deformation is entirely dissipated into heat. Hence, the dissipated work per unit volume is equal to the plastic work

$$\frac{dW^{\mathrm{diss}}}{dt} = \frac{dW^{\mathrm{p}}}{dt} = \sigma\dot{\varepsilon}^{p} \tag{5}$$

For dislocation plasticity, this statement needs to be qualified: strain hardening is associated with the change of an internal variable (the dislocation density). Since dislocations carry

elastic energy in form of stress and strain fields and the much smaller contribution from the dislocation core energy, this leads to a stored internal energy contribution (a stored defect energy[4]). We estimate the defect energy storage rate as

$$\frac{dE^{\text{def}}}{dt} = E_L \frac{d\rho}{d\varepsilon} \dot{\varepsilon}^p \tag{6}$$

where E_L is the dislocation line energy. To obtain an upper estimate of the dislocation storage rate, we use the well-established Kocks-Mecking model and note that an upper bound to the dislocation storage rate is obtained by neglecting dynamic recovery. In that case, following Supplementary Ref. [5] and using $E_{\text{L}} = \lambda G b^2$, we can estimate

$$\left(\frac{\alpha G b}{2}\right)^2 \frac{\partial \rho}{\partial \varepsilon^{\text{p}}} = \sigma \frac{\partial \sigma}{\partial \varepsilon^{\text{p}}} \le \sigma \theta_{\text{II}}, \quad \frac{dE^{\text{def}}}{dt} \le \frac{\theta_{\text{II}}}{G}\left(\frac{4\eta}{\alpha^2}\right)\sigma \dot{\varepsilon}^p = \frac{\theta_{\text{II}}}{G}\left(\frac{4\lambda}{\alpha^2}\right)\frac{dW^p}{dt} \tag{7}$$

where θ_{II} is the initial hardening slope in the limit of athermal strain rate independent deformation (in FCC single crystals: hardening stage II). The numerical factor in the bracket on the right-hand side of Supplementary Eq. (7) is of the order of 1. Since the hardening slope is only a tiny fraction of the shear modulus ($\theta_{\text{II}} \approx G/200$ [5]), the defect energy storage rate is only a tiny fraction of the plastic work rate. Hence Supplementary Eq. (5) holds for dislocation plasticity with only minor corrections due to stored defect energy.

On the dislocation level, dissipation occurs because the work that is expended in moving dislocations is dissipated into the phonon system. The situation is particularly simple for non-relativistic over-damped dislocation motion, because there, due to absence of inertia, all work is instantaneously dissipated. Using $v = (b/B)\tau$ and $F^{PK} = b\tau$ we write the dissipated energy as

$$\frac{dW^{\text{diss}}}{dt} = \frac{1}{V}\int_{(\mathbb{C}_V)} F^{\text{PK}} v\, ds = \frac{B}{V}\int_{(\mathbb{C}_V)} v^2 ds \tag{8}$$

where the integral runs over the set $\mathbb{C}_V$ of all dislocation lines in the volume V considered. Introducing the dislocation density and the mean square dislocation velocity as

$$\rho = \frac{1}{V}\int_{(\mathbb{C}_V)} ds \quad , \quad \langle v^2 \rangle = \frac{\int_{(\mathbb{C}_V)} v^2 ds}{\int_{(\mathbb{C}_V)} ds} \tag{9}$$

we thus find that the mean velocity square is related to the microscopically dissipated work by

$$\frac{dW^{\text{diss}}}{dt} = B\rho\langle v^2 \rangle \quad . \tag{10}$$

It is clear that the microscopically dissipated work and the macroscopic dissipated work must be identical. We therefore obtain a relationship between the 'macroscopic' quantities in Supplementary Eq. (5) and the 'microscopic' quantities in Supplementary Eq. (10):

$$\frac{dW^{\text{diss}}}{dt} = B\rho\langle v^2 \rangle = \sigma\dot{\varepsilon}^p \quad . \tag{11}$$

We write the plastic strain rate now in terms of microscopic quantities (segment velocities) as

$$\dot{\varepsilon}^p = \frac{b}{V}\int_{(\mathbb{C}_V)} m(s)v(s)ds =: m\rho b\langle v \rangle \quad , \quad \langle v \rangle = \frac{\int_{(\mathbb{C}_V)} m(s)vds}{m\int_{(\mathbb{C}_V)} ds} \quad , \tag{12}$$

where we note that the motion of dislocations on inactive slip systems (Schmidt factor: $m(s)=0$) does not contribute to the m-weighted average velocity. Accordingly, this average velocity $\langle v \rangle$ of all dislocations relates to the mean velocity v_{m} on the active slip systems via $\langle v \rangle = f_{\text{a}} v_{\text{m}}$.

Using Eq. (3a) of the main paper, we find $\sigma = m^{-1}(\alpha G b\sqrt{\rho} + (1/f_{\text{a}})(B/b)\langle v \rangle)$. This leads to our final result,

$$\frac{\langle v^2 \rangle}{\langle v \rangle^2} = \frac{\alpha G b^3}{B}\left(\frac{\rho}{\dot{\varepsilon}^{2/3}}\right)^{3/2} + \frac{1}{f_a} = \frac{1}{f_a}\left(\alpha \mathrm{P}^{3/2} + 1\right) \quad . \tag{13}$$

We can envisage Supplementary Eq. (13) as an expression for the magnitude of fluctuations. We define the weighted coefficient of variation of dislocation velocities as

$$\mathrm{COV}_M = \frac{\left\langle \delta v^2 \right\rangle^{0.5}}{\langle v \rangle} = \left(\frac{\left\langle v^2 \right\rangle}{\langle v \rangle^2} - 1 \right)^{1/2} = \frac{\alpha G b^3}{B} \left(\frac{\rho}{\dot{\varepsilon}^{2/3}} \right)^{3/4} \quad . \tag{14}$$

This quantity measures the magnitude of dislocation velocity fluctuations. As we move to very small strain rates, this quantity diverges, which indicates a critical behavior. We note that the general idea of the above derivation goes back to Hähner[6] and the case of a linear drag law has, in embryonic form, been previously considered by one of the present authors[7].

Supplementary Note 3: Data analysis

Comparison of our theoretical predictions and simulation data with experimental data (Fig. 3 in the main paper) is somewhat hampered by the fact that a meaningful comparison requires, on the experimental side, data where the strain rate, dislocation density, and yield/flow stress have been determined simultaneously. (Note that, in view of our discussion of yield plastic strains in Supplementary Note 1, it is not necessary that the data have been determined at 0.2% plastic strain as long as the strain rate/dislocation density combination falls into the forest hardening or drag controlled regimes. Nevertheless, we have mostly considered experimental data obtained at low strains (less than 5%), since such low strains prevent the development of strongly heterogeneous dislocation structures such as cell or subgrain patterns.)

Even if the 0.2% plastic strain definition of the yield stress is relaxed, experimental data of sufficient quality are comparatively scarce in the literature, since a reliable determination of dislocation densities requires a significant experimental effort. Moreover, while in simulations it is easy to determine dislocation densities pertaining to a given stress and plastic strain in the loaded state, in experiments this is exceedingly difficult. As a consequence, most experiments report the initial dislocation density before loading or the dislocation density after unloading. It is expected that such data somewhat underestimate the actual dislocation density under load, since bowed-out dislocations relax into stable configurations, see our discussion of ρ_0 vs ρ_y in the main paper.

These difficulties imply that, in selecting experimental data to substantiate our theoretical conjectures, certain compromises are inevitable. For instance, experimental studies published

in the literature may provide the initial dislocation densities of single crystal specimens and the flow stresses at a finite strain level. We have included such data if stresses were measured at strains less than 5%, because, in FCC single crystals, the strain hardening rate after the onset of deformation is low (hardening stage I, easy glide)[8] and the dislocation density increases at most by a factor of 2 below a strain of 5% in Cu[9] and Al[10]. In Table 1 of Supplementary Data 2, detailed information regarding the measurement methods of stress and dislocation density and the data source is provided for all the experimental studies cited in Fig. 3 of the main paper.

In addition to the experimental works cited in the main paper, there exist numerous published works which qualitatively support our main findings. For instance, the yield/flow stress decreases and then increases as the pre-strain increases, as reported in multiple experimental studies on Cu[11] and LiF[12,13]. We have not included these and similar studies in the main paper unless they contain explicit dislocation density data, which allow for quantitative or at least semi-quantitative comparison with our model.

To ensure that the simulations, which we use to develop and support our material strength model, are not beset by artifacts of the simulation code, we employed two completely independent simulation methods (MD vs ParaDiS DDD). In addition, in Fig. 3 of the main paper, which shows the essential results of our work, we have incorporated, in support of our findings, published DDD simulation results by other groups who use different DDD codes and/or different simulation settings (initial configurations, boundary conditions, yield/flow stress definitions). The comparison with our own simulation data and model predictions serves to underpin the robustless of our findings. A compilation of the data sources and main settings of these simulations is provided in Table 2 of Supplementary Data 2.

A complete compilation of our own yield stress data used in Fig. 3 of the main paper is provided in Table 3 of Supplementary Data 2, while all stress-strain curves are compiled in Supplementary Figure 4 and typical dislcoation configurations of the DDD and MD simulations are shown in Supplementary Figures 5 and 6, respectively.

Supplementary Note 4: Statistical analysis of local plastic strain distribution

For the simulation depicted in Fig. 4i of the main paper, we conducted a statistical

analysis of the plastic strain distribution. The simulation cell is divided into 64000 sub-elements. Then, in Supplementary Fig. 3, the probability density is plotted as a function of normalized plastic strain at different total plastic strain levels. In Supplementary Fig. 3, below the total plastic strain of 0.2%, the distribution does not change much. For total plastic strain > 0.2%, we can see two humps appear on the curves. The second hump corresponds to the slip plane with the largest plastic strain, while the first hump corresponds to other slip planes. Both humps increase in height and move rightwards as the total plastic strain increases, indicating that the plastic strain localizes more significantly. Therefore, the influence of plastic strain localization on dislocation plasticity becomes stronger as the total plastic strain increases. However, at the total plastic yield strain of 0.2%, which is considered in the current work, the influence of plastic strain localization on the material strength is still slight.

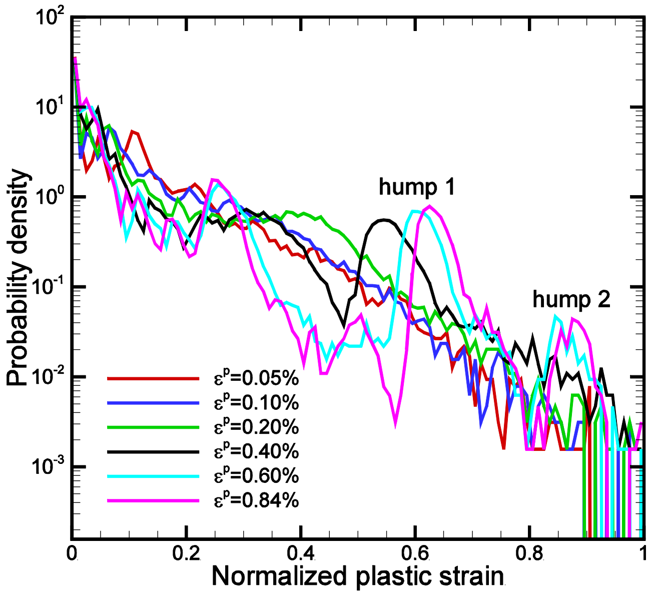

Supplementary Figure 3. Statistics of local plastic strain at different total plastic strains. $\rho_0=6.8\times10^{11}m^{-2}$ and $\dot{\varepsilon}=10^6s^{-1}$. The final strain pattern in this simulation is shown in Fig. 4i of the main paper.

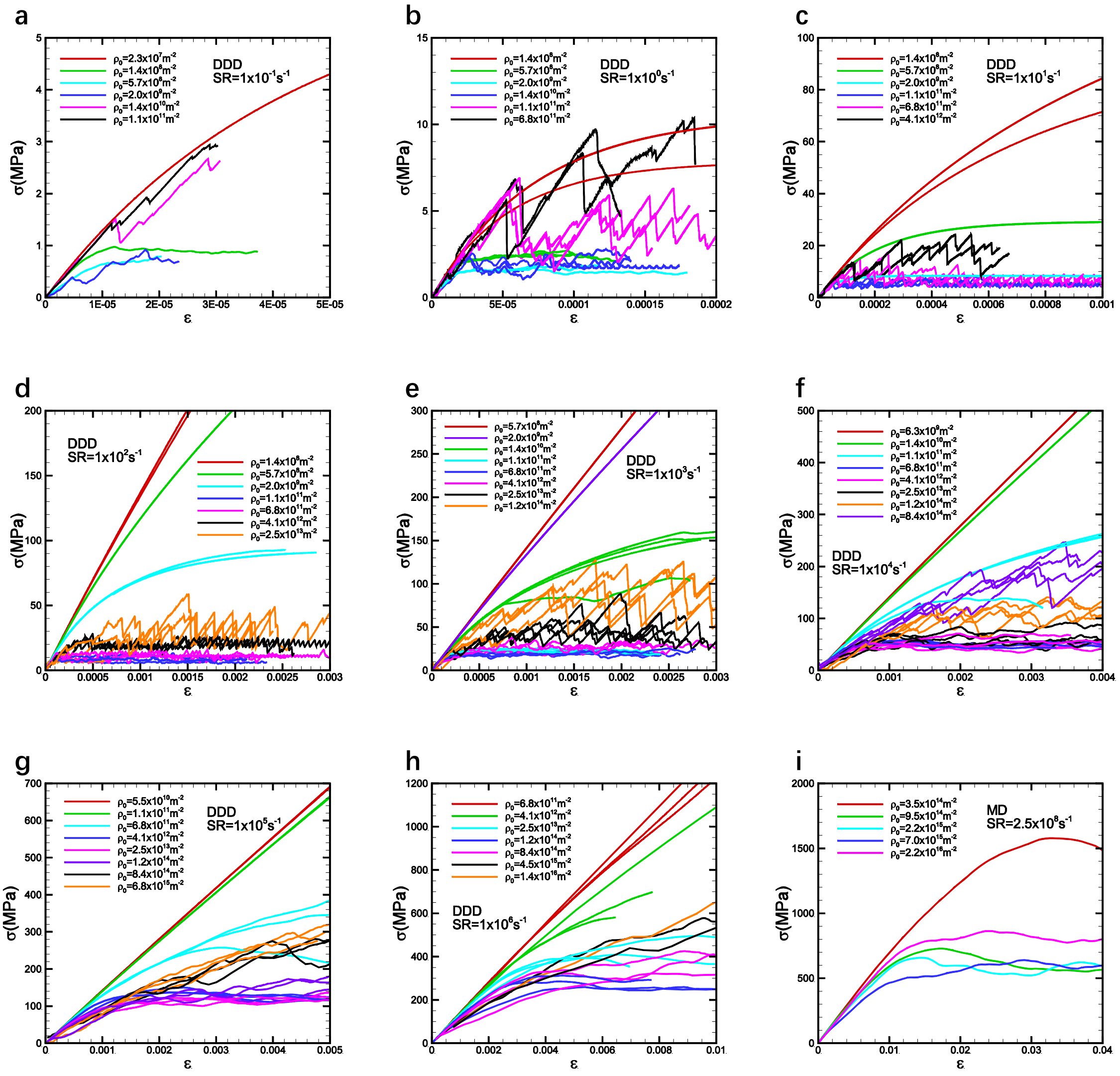

Supplementary Figure 4. All the stress-strain curves from our DDD and MD simulations. a. SR=10^{-1}s^{-1}; **b**. SR=10^{0}s^{-1}; **c** SR=10^{1}s^{-1}; **d**. SR=10^{2}s^{-1}; **e**. SR=10^{3}s^{-1}; **f**. SR=10^{4}s^{-1}; **g**. SR=10^{5}s^{-1}; **h**. SR=10^{6}s^{-1}; **i**. SR=2.5×10^{8}s^{-1}. DDD is abbreviation of discrete dislocation dynamics and MD is molecular dynamics. SR is abbreviation of strain rate.

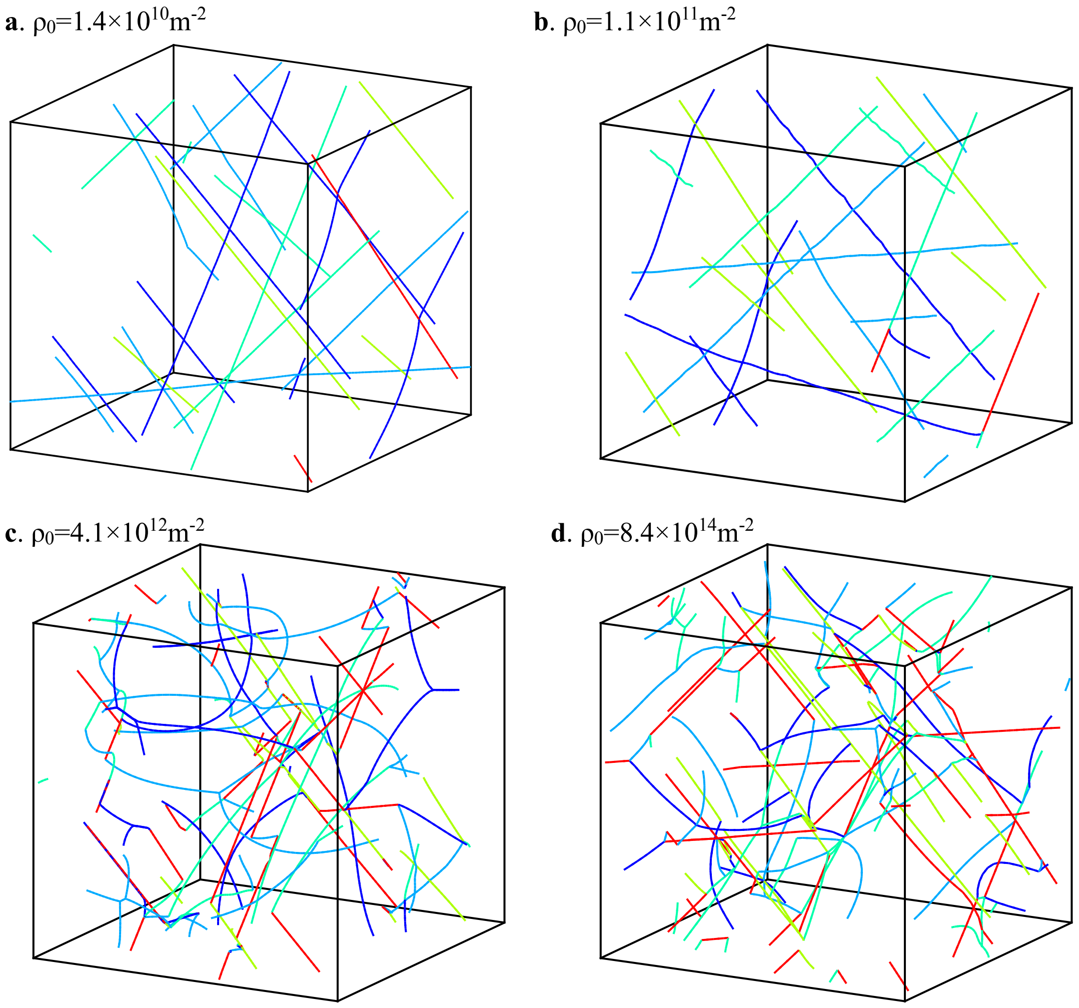

Supplementary Figure 5. Dislocation configurations of DDD simulations at 0.2% plastic strain and strain rate 10^4 s^{-1}. **a**. ρ_0=1.4×10^{10}m^{-2}; **b**. ρ_0=1.1×10^{11}m^{-2}; **c**. ρ_0=4.1×10^{12}m^{-2}; **d**. ρ_0=8.4×10^{14}m^{-2}. Note that the simulation cell size changes as the initial dislocation density increases. DDD is abbreviation of discrete dislocation dynamics.

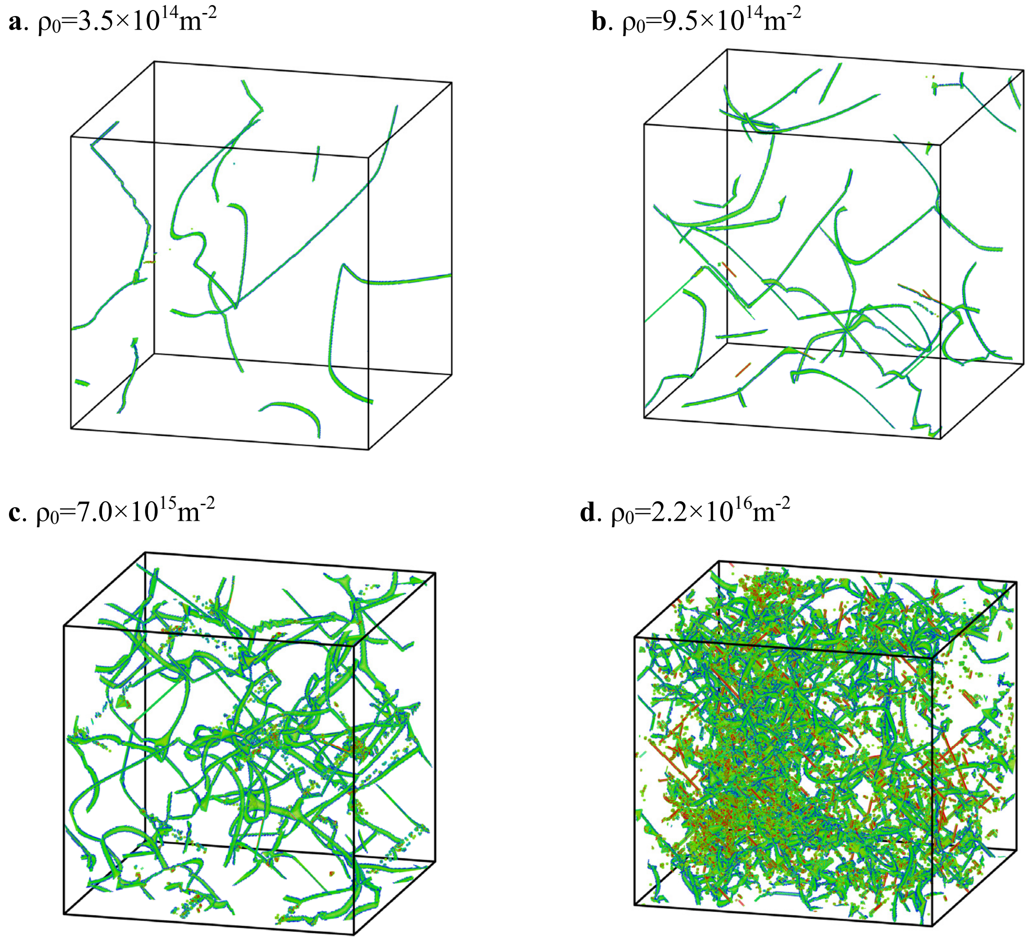

Supplementary Figure 6. Dislocation configurations of MD simulations at 0.2% plastic strain and strain rate 2.5×10^{8} s^{-1}. **a**. ρ_0=3.5×10^{14}m^{-2}; **b**. ρ_0=9.5×10^{14}m^{-2}; **c**. ρ_0=7.0×10^{15}m^{-2}; **d**. ρ_0=2.2×10^{16}m^{-2}. MD is abbreviation of molecular dynamics.

Supplementary References

1 Zaiser, M. & Sandfeld, S. Scaling properties of dislocation simulations in the similitude regime. *Modelling and Simulation in Materials Science and Engineering* **22**, 065012 (2014).

2 Hirth, J. P. & Lothe, J. *Theory of Dislocations*. 2nd edn, (John Wiley and Sons, 1982).

3 Khraishi, T. A., Zbib, H. M., de la Rubia, T. D. & Victoria, M. Localized deformation and hardening in irradiated metals: Three-dimensional discrete dislocation dynamics simulations. *Metallurgical and Materials Transactions B* **33**, 285-296 (2002).

4 Zaiser, M. Local density approximation for the energy functional of three-dimensional dislocation systems. *Physical Review B* **92**, 174120 (2015).

5 Mecking, H. & Kocks, U. F. Kinetics of flow and strain-hardening. *Acta Metall.* **29**, 1865-1875 (1981).

6 Hähner, P. On the foundations of stochastic dislocation dynamics. *Appl. Phys. A* **62**, 473-481 (1996).

7 Zaiser, M. Statistical modelling of dislocation systems. *Mater. Sci. Eng. A* **309-310**, 304-315 (2001).

8 Thornton, P. R., Mitchell, T. E. & Hirsch, P. B. The strain-rate dependence of the flow stress of copper single crystals. *The Philosophical Magazine: A Journal of Theoretical Experimental and Applied Physics* **7**, 337-358 (1962).

9 Edington, J. W. The influence of strain rate on the mechanical properties and dislocation substructure in deformed copper single crystals. *The Philosophical Magazine: A Journal of Theoretical Experimental and Applied Physics* **19**, 1189-1206 (1969).

10 Chiem, C. Y. & Duffy, J. Strain rate history effects and observations of dislocation substructure in aluminum single crystals following dynamic deformation. *Mater. Sci. Eng.* **57**, 233-247 (1983).

11 Jones, O. E. & Mote, J. D. Shock-Induced Dynamic Yielding in Copper Single Crystals. *Journal of Applied Physics* **40**, 4920-4928 (1969).

12 Johnston, W. G. Yield Points and Delay Times in Single Crystals. *Journal of Applied Physics* **33**, 2716-2730 (1962).

13 Haworth, W. L., Davis, L. A. & Gordon, R. B. Pressure Dependence of the Mobility of Dislocations in Lithium Fluoride Crystals. *Journal of Applied Physics* **39**, 3818-3821 (1968).
