# Supplementary material for: Strain rate dependency of dislocation plasticity
Source: Nat Commun. 2021 Mar 23;12:1845. doi: 10.1038/s41467-021-21939-1 (PMC7988163; doi:10.1038/s41467-021-21939-1)
Supplement: Supplementary file 2 — Description of Additional Supplementary Files [file 41467_2021_21939_MOESM2_ESM.pdf]

Description of Additional Supplementary Files

File Name: Supplementary Data 1
Description: DDD/MD Simulation data

File Name: Supplementary Data 2
Description: Data of Fig. 3 of main paper

File Name: Supplementary Movie 1
Description: Dislocation evolution at strain rate of $10^6 s^{-1}$

File Name: Supplementary Movie 2
Description: Dislocation evolution at strain rate of $10^4 s^{-1}$

File Name: Supplementary Movie 3
Description: Dislocation evolution at strain rate of $10^2 s^{-1}$
