# Supplementary material for: Strain rate dependency of dislocation plasticity
Source: Nat Commun. 2021 Mar 23;12:1845. doi: 10.1038/s41467-021-21939-1 (PMC7988163; doi:10.1038/s41467-021-21939-1)
Supplement: Supplementary file 3 — Supplementary Data 1 [file 41467_2021_21939_MOESM3_ESM.zip › Supplementary Data 1/Note.docx]

1. The datasets belong to the paper: Strain rate dependency of dislocation plasticity, Haidong Fan, Qingyuan Wang, Jaafar A. El-Awady, Dierk Raabe, Michael Zaiser, Nature Communications, 2021.
2. The datasets include 189 DDD (discrete dislocation dynamics) simulations and 5 MD (molecular dynamics) simulations on the effects of dislocation density (from 2.3×10^7^ m^-2^ to 2.2×10^16^ m^-2^) and strain rate (0.1 s^-1^ to 2.5×10^8^ s^-1^) on the material strength of bulk copper and aluminum single-crystals.
3. In the DDD simulations, the stress, dislocation density, plastic strain, and mean dislocation velocity are shown as functions of the loading strain. Note that the data during the initial relaxation process are not shown since no data on loading strain are available. So the initial dislocation density shown in the file name does not correspond to the first raw in that datafile.
4. In the MD simulations, only the stress-strain data are given since other information is not directly available.
