# Supplementary material for: Strain rate dependency of dislocation plasticity
Source: Nat Commun. 2021 Mar 23;12:1845. doi: 10.1038/s41467-021-21939-1 (PMC7988163; doi:10.1038/s41467-021-21939-1)
Supplement: Supplementary file 4 — Supplementary Data 2 [file 41467_2021_21939_MOESM4_ESM.zip › Supplementary_Data_2.docx]

**Strain rate dependency of dislocation plasticity**

Haidong Fan^1,2*^, Qingyuan Wang^1*^, Jaafar A. El-Awady^3^, Dierk Raabe^2^, Michael Zaiser^4^

^1^ Department of Mechanics, Sichuan University, Chengdu 610065, China

^2^ Department Microstructure Physics and Alloy Design, Max-Planck-Institut für Eisenforschung GmbH, Düsseldorf 40237, Germany

^3^ Department of Mechanical Engineering, Whiting School of Engineering, The Johns Hopkins University, Baltimore, MD 21218, USA

^4^ WW8-Materials Simulation, Department of Materials Science, FAU Universität Erlangen-Nürnberg, Fürth 90762, Germany

*Corresponding authors emails: hfan85@scu.edu.cn (HF), wangqy@scu.edu.cn (QW)

Table 1. Experimental data published in the literature that have been used in Fig. 3 of the main paper.

| Strain rate (s^-1^) | Dislocation density (m^-2^) | Shear stress (MPa) | Material | Data source and note |
| --- | --- | --- | --- | --- |
| 1.70E-05 | 2.05E+10 | 6.35E-01 | Cu | Shear stresses are picked up from the stress-strain curves at different strains < 10%.  Dislocation densities are determined by etch pit method after unloading of the deformed samples.  Data are from Fig. 4 in Ref. [^1^](#_ENREF_1). |
| 1.70E-05 | 2.94E+10 | 1.75E+00 |  |  |
| 1.70E-05 | 4.00E+10 | 1.73E+00 |  |  |
| 1.70E-05 | 7.20E+10 | 9.81E-01 |  |  |
| 1.70E-05 | 1.14E+11 | 1.32E+00 |  |  |
| 1.70E-05 | 1.22E+11 | 1.56E+00 |  |  |
| 1.70E-05 | 7.56E+10 | 3.23E+00 |  |  |
| 1.70E-05 | 7.52E+10 | 3.59E+00 |  |  |
| 1.70E-05 | 1.19E+11 | 3.30E+00 |  |  |
| 1.70E-05 | 1.36E+11 | 2.87E+00 |  |  |
| 1.70E-05 | 1.99E+11 | 2.70E+00 |  |  |
| 1.70E-05 | 2.03E+11 | 5.29E+00 |  |  |
| 1.70E-05 | 2.24E+11 | 4.59E+00 |  |  |
| 1.70E-05 | 3.13E+11 | 4.40E+00 |  |  |
| 1.70E-05 | 3.56E+11 | 5.16E+00 |  |  |
| 1.70E-05 | 4.80E+11 | 7.44E+00 |  |  |
| 1.70E-05 | 6.13E+11 | 4.82E+00 |  |  |
| 1.70E-05 | 1.54E+12 | 7.63E+00 |  |  |
| 6.30E-05 | 9.56E+05 | 9.99E-01 | CuNi | Shear stresses are the critical resolved shear stresses at stage I.  Dislocation densities are the initial dislocation densities (determined by etch pit method before loading).  Data are from Fig. 4 in Ref. [^2^](#_ENREF_2). |
| 6.30E-05 | 8.95E+06 | 5.45E-01 |  |  |
| 6.30E-05 | 1.58E+07 | 7.20E-01 |  |  |
| 6.30E-05 | 1.61E+07 | 5.45E-01 |  |  |
| 6.30E-05 | 2.80E+07 | 5.19E-01 |  |  |
| 6.30E-05 | 1.01E+08 | 6.44E-01 |  |  |
| 6.30E-05 | 1.82E+09 | 1.18E+00 |  |  |
| 6.30E-05 | 2.29E+09 | 1.31E+00 |  |  |
| 6.30E-05 | 2.11E+09 | 1.39E+00 |  |  |
| 6.30E-05 | 3.08E+09 | 1.50E+00 |  |  |
| 6.30E-05 | 5.56E+09 | 1.62E+00 |  |  |
| 6.30E-05 | 1.85E+10 | 1.97E+00 |  |  |
| 6.30E-05 | 1.41E+09 | 6.49E-01 | Cu | Stresses are the critical resolved shear stresses at stage I.  Dislocation densities are the initial dislocation densities (determined by etch pit method before loading).  Data are from Fig. 4 in Ref. [^2^](#_ENREF_2). |
| 6.30E-05 | 1.59E+09 | 5.95E-01 |  |  |
| 6.30E-05 | 1.92E+09 | 6.16E-01 |  |  |
| 6.30E-05 | 2.03E+09 | 7.19E-01 |  |  |
| 6.30E-05 | 2.67E+09 | 7.43E-01 |  |  |
| 6.30E-05 | 3.13E+09 | 7.40E-01 |  |  |
| 6.30E-05 | 4.13E+09 | 7.22E-01 |  |  |
| 6.30E-05 | 5.27E+09 | 8.31E-01 |  |  |
| 6.30E-05 | 6.73E+09 | 8.49E-01 |  |  |
| 6.30E-05 | 8.43E+09 | 9.36E-01 |  |  |
| 6.30E-05 | 1.78E+10 | 8.97E-01 |  |  |
| 6.30E-05 | 1.42E+10 | 1.02E+00 |  |  |
| 6.30E-05 | 7.64E+10 | 1.32E+00 |  |  |
| 6.30E-05 | 7.92E+10 | 1.38E+00 |  |  |
| 1.00E-04 | 1.96E+10 | 1.02E+00 | Cu | Resolved shear stresses are calculated from the flow stresses before unloading.  Dislocation densities are measured by etch pit method performed on [111] oriented sections after unloading.  Data are from Fig. 2 in Ref. [^3^](#_ENREF_3). |
| 1.00E-04 | 3.32E+10 | 1.03E+00 |  |  |
| 1.00E-04 | 5.92E+10 | 1.58E+00 |  |  |
| 1.00E-04 | 8.82E+10 | 2.51E+00 |  |  |
| 1.00E-04 | 2.87E+11 | 4.52E+00 |  |  |
| 1.00E-04 | 3.91E+11 | 5.54E+00 |  |  |
| 1.00E-04 | 4.56E+11 | 3.86E+00 |  |  |
| 1.00E-04 | 4.92E+11 | 5.54E+00 |  |  |
| 1.00E-04 | 1.22E+12 | 7.92E+00 |  |  |
| 1.00E-04 | 3.96E+12 | 1.54E+01 |  |  |
| 1.00E-04 | 6.25E+12 | 1.95E+01 |  |  |
| 1.00E-04 | 8.42E+12 | 2.61E+01 |  |  |
| 1.00E-04 | 1.24E+13 | 2.84E+01 |  |  |
| 1.00E-04 | 1.41E+13 | 2.78E+01 |  |  |
| 1.00E-04 | 2.87E+13 | 3.17E+01 |  |  |
| 1.00E-04 | 3.49E+13 | 4.00E+01 |  |  |
| 1.00E-04 | 2.21E+14 | 1.13E+02 |  |  |
| 1.00E-04 | 1.00E+08 | 6.60E-01 | Cu | Resolved shear stresses are calculated from the macroscopic yield stresses defined by change in slope of load-elongation curve.  Dislocation densities are the initial dislocation densities determined by etch pit method.  Data are from Table 1 in Ref. [^4^](#_ENREF_4). |
| 1.00E-04 | 1.00E+08 | 3.70E-01 |  |  |
| 1.00E-04 | 2.00E+08 | 3.70E-01 |  |  |
| 1.00E-04 | 2.00E+08 | 3.50E-01 |  |  |
| 1.00E-04 | 1.50E+09 | 3.40E-01 |  |  |
| 1.00E-04 | 1.00E+08 | 3.80E-01 |  |  |
| 1.00E-04 | 2.00E+09 | 3.70E-01 |  |  |
| 1.00E-04 | 1.00E+10 | 9.40E-01 |  |  |
| 6.00E-04 | 1.57E+08 | 5.90E+01 | Si | Resolved shear stresses are calculated from the upper yield stresses.  Dislocation densities are determined before deformation using etch pit method on [111] surface.  Data are from Fig. 6 in Ref. [^5^](#_ENREF_5). |
| 6.00E-04 | 1.57E+08 | 5.03E+01 |  |  |
| 6.00E-04 | 1.42E+09 | 3.85E+01 |  |  |
| 6.00E-04 | 3.96E+09 | 2.88E+01 |  |  |
| 6.00E-04 | 9.34E+09 | 2.07E+01 |  |  |
| 6.00E-04 | 1.78E+10 | 1.67E+01 |  |  |
| 6.00E-04 | 4.33E+10 | 1.61E+01 |  |  |
| 4.00E+02 | 2.20E+10 | 9.73E+00 | LiF | Resolved shear stresses are calculated from the upper yield stresses after strain rate change.  Dislocation densities are measured by etch pit method before the strain rate change at 0.5%, 1%, 1.5%, 1.8% strains.  Data are from Fig. 4 and Table 1 in Ref. [^6^](#_ENREF_6). |
| 4.00E+02 | 4.70E+10 | 8.78E+00 |  |  |
| 4.00E+02 | 1.20E+11 | 7.51E+00 |  |  |
| 4.00E+02 | 1.40E+11 | 8.10E+00 |  |  |
| 4.00E+02 | 1.50E+11 | 8.03E+00 |  |  |
| 8.00E+02 | 2.20E+10 | 1.21E+01 |  |  |
| 8.00E+02 | 4.70E+10 | 1.00E+01 |  |  |
| 8.00E+02 | 1.20E+11 | 9.10E+00 |  |  |
| 8.00E+02 | 1.40E+11 | 9.32E+00 |  |  |
| 4.37E-05 | 4.70E+10 | 1.63E+00 |  | Resolved shear stresses are at 0.5% strain.  Dislocation densities are same as above.  Data are from Fig. 5 and Table 1 in Ref. [^6^](#_ENREF_6). |
| 1.76E-04 | 4.70E+10 | 1.73E+00 |  |  |
| 6.87E+02 | 4.70E+10 | 1.04E+01 |  |  |
| 4.28E-05 | 1.40E+11 | 3.19E+00 |  | Resolved shear stresses are at 1.5% strain.  Dislocation densities are same as above.  Data are from Fig. 5 and Table 1 in Ref. [^6^](#_ENREF_6). |
| 1.62E-04 | 1.40E+11 | 3.33E+00 |  |  |
| 4.14E+02 | 1.40E+11 | 9.69E+00 |  |  |
| 7.19E+02 | 1.40E+11 | 1.23E+01 |  |  |
| 4.29E-05 | 4.16E+12 | 2.12E+00 | Al | Resolved shear stresses are at shear strain of 1.5%.  Dislocation densities are measured by TEM after 1.5% shear strain.  Data are from Fig. 4 and Fig. 8 in Ref. [^7^](#_ENREF_7). |
| 5.47E-04 | 4.16E+12 | 2.38E+00 |  |  |
| 5.09E+02 | 4.16E+12 | 2.93E+00 |  |  |
| 7.99E+02 | 4.16E+12 | 3.21E+00 |  |  |
| 9.60E+02 | 4.16E+12 | 3.50E+00 |  |  |
| 1.16E+03 | 4.16E+12 | 4.05E+00 |  |  |
| 1.40E+03 | 4.16E+12 | 4.44E+00 |  |  |
| 8.50E+02 | 4.96E+12 | 2.85E+00 |  | Resolved shear stresses are measured when the samples are reloaded after different pre-strains.  Dislocation densities are measured by TEM after same pre-strains.  Data are from Fig. 8 and Table 2 in Ref. [^7^](#_ENREF_7). |
| 8.50E+02 | 5.77E+12 | 4.10E+00 |  |  |
| 8.50E+02 | 8.29E+12 | 6.23E+00 |  |  |
| 8.50E+02 | 1.19E+13 | 7.82E+00 |  |  |
| 8.50E+02 | 1.78E+13 | 8.88E+00 |  |  |
| 3.83E+03 | 6.20E+12 | 6.94E+00 | Al | Shear stresses are at shear strain of 1%.  Dislocation densities are calculated from Saada theory.  Data are from Fig. 2 and Table 1 in Ref. [^8^](#_ENREF_8). |
| 4.16E+03 | 6.20E+12 | 7.78E+00 |  |  |
| 1.24E+04 | 6.20E+12 | 1.79E+01 |  |  |
| 1.88E+04 | 6.20E+12 | 2.36E+01 |  |  |
| 1.82E+04 | 6.20E+12 | 2.39E+01 |  |  |
| 2.67E+04 | 6.20E+12 | 3.27E+01 |  |  |
| 3.83E+03 | 6.20E+12 | 6.94E+00 |  |  |
| 4.09E-04 | 6.05E+12 | 6.62E+00 | Cu | Resolved shear stresses are at 0.1% plastic strain.  Dislocation densities are measured at the easy glide stage.  Data are from Fig. 2 and Fig. 9 in Ref. [^9^](#_ENREF_9). |
| 4.20E-03 | 6.05E+12 | 6.28E+00 |  |  |
| 4.41E-02 | 6.05E+12 | 6.62E+00 |  |  |
| 1.99E+00 | 6.05E+12 | 7.25E+00 |  |  |
| 1.01E+01 | 6.05E+12 | 7.88E+00 |  |  |
| 1.31E+01 | 6.05E+12 | 7.59E+00 |  |  |
| 7.95E+01 | 6.05E+12 | 7.88E+00 |  |  |
| 3.15E+02 | 6.05E+12 | 1.39E+01 |  |  |
| 3.79E+02 | 6.05E+12 | 1.14E+01 |  |  |
| 1.92E+03 | 6.05E+12 | 2.36E+01 |  |  |
| 1.92E+03 | 6.05E+12 | 2.40E+01 |  |  |
| 3.45E+03 | 6.05E+12 | 5.51E+01 |  |  |
| 4.49E+03 | 6.05E+12 | 5.79E+01 |  |  |
| 4.87E+03 | 6.05E+12 | 5.91E+01 |  |  |
| 7.01E+03 | 6.05E+12 | 7.36E+01 |  |  |
| 5.83E+03 | 6.05E+12 | 7.42E+01 |  |  |
| 7.01E+03 | 6.05E+12 | 7.49E+01 |  |  |
| 7.30E+03 | 6.05E+12 | 8.93E+01 |  |  |
| 1.30E+07 | 6.77E+13 | 4.86E+02 | Cu | Shear stress is calculated from the flow stress.  Dislocation density is calculated from theoretical analysis.  Data are from Fig. 17 and Fig. 18 in Ref. [^10^](#_ENREF_10). |

Table 2. DDD simulation data from published literature used in Fig. 3 of the main paper.

| Strain rate  (s^-1^) | Dislocation density (m^-2^) | Shear stress (MPa) | Material | Data source and note |
| --- | --- | --- | --- | --- |
| 1.00E+06 | 1.50E+12 | 1.29E+03 | Cu | Shear stress is calculated from the peak stress.  Dislocation density is the initial dislocation density.  Data are from Fig. 4 in Ref. [^11^](#_ENREF_11). |
| 1.00E+02 | 6.68E+12 | 1.54E+01 | Cu | Stresses are measured at strain of 0.2-0.8%.  Dislocation densities are measured at 0.1% plastic strain.  Data are from Fig. 2 and Fig. 12 in Ref. [^12^](#_ENREF_12). |
| 1.00E+03 | 6.68E+12 | 1.73E+01 |  |  |
| 1.00E+04 | 6.68E+12 | 2.37E+01 |  |  |
| 1.00E+05 | 6.68E+12 | 7.55E+01 |  |  |
| 1.00E+06 | 6.68E+12 | 2.74E+02 |  |  |

Table 3. Simulation data of the present authors shown in Fig. 3 in the main paper.

| Strain rate  $\dot{\varepsilon}$ (s^-1^) | Dislocation density at yield, ρ_y_ (m^-2^) | Yield stress τ_y_ (MPa) | Yield plastic strain $\varepsilon_{y}^{p}$ | Material and method |
| --- | --- | --- | --- | --- |
| 1.00E-01 | 2.26E+07 | 1.38E+00 | 1.01E-05 | DDD, Cu |
| 1.00E-01 | 1.73E+08 | 3.81E-01 | 1.00E-05 |  |
| 1.00E-01 | 5.83E+08 | 2.81E-01 | 1.00E-05 |  |
| 1.00E-01 | 1.98E+09 | 3.05E-01 | 1.00E-05 |  |
| 1.00E-01 | 1.47E+10 | 1.08E+00 | 1.00E-05 |  |
| 1.00E-01 | 1.10E+11 | 1.19E+00 | 1.00E-05 |  |
| 1.00E+00 | 1.42E+08 | 3.86E+00 | 1.00E-04 |  |
| 1.00E+00 | 1.10E+09 | 9.31E-01 | 1.00E-04 |  |
| 1.00E+00 | 3.03E+09 | 7.47E-01 | 1.00E-04 |  |
| 1.00E+00 | 1.39E+10 | 8.82E-01 | 1.00E-04 |  |
| 1.00E+00 | 1.07E+11 | 1.95E+00 | 1.00E-04 |  |
| 1.00E+00 | 7.19E+11 | 3.38E+00 | 1.00E-04 |  |
| 1.00E+00 | 1.41E+08 | 3.00E+00 | 1.01E-04 |  |
| 1.00E+00 | 1.03E+09 | 1.06E+00 | 8.09E-05 |  |
| 1.00E+00 | 3.03E+09 | 7.47E-01 | 1.00E-04 |  |
| 1.00E+00 | 1.38E+10 | 7.47E-01 | 1.00E-04 |  |
| 1.00E+00 | 1.08E+11 | 1.62E+00 | 1.00E-04 |  |
| 1.00E+00 | 1.41E+08 | 3.86E+00 | 1.00E-04 |  |
| 1.00E+00 | 1.55E+09 | 9.43E-01 | 1.00E-04 |  |
| 1.00E+00 | 2.03E+09 | 5.88E-01 | 1.00E-04 |  |
| 1.00E+00 | 1.45E+10 | 1.11E+00 | 1.00E-04 |  |
| 1.00E+00 | 1.04E+11 | 1.51E+00 | 1.00E-04 |  |
| 1.00E+00 | 7.33E+11 | 1.92E+00 | 1.00E-04 |  |
| 1.00E+01 | 1.41E+08 | 3.68E+01 | 5.00E-04 |  |
| 1.00E+01 | 5.66E+08 | 1.16E+01 | 5.00E-04 |  |
| 1.00E+01 | 1.96E+09 | 3.41E+00 | 5.01E-04 |  |
| 1.00E+01 | 1.22E+11 | 3.28E+00 | 5.00E-04 |  |
| 1.00E+01 | 6.61E+11 | 2.82E+00 | 5.00E-04 |  |
| 1.00E+01 | 4.11E+12 | 6.98E+00 | 5.00E-04 |  |
| 1.00E+01 | 1.41E+08 | 2.91E+01 | 5.01E-04 |  |
| 1.00E+01 | 5.66E+08 | 1.15E+01 | 5.01E-04 |  |
| 1.00E+01 | 1.96E+09 | 3.38E+00 | 5.01E-04 |  |
| 1.00E+01 | 2.54E+10 | 2.09E+00 | 3.78E-04 |  |
| 1.00E+01 | 1.06E+11 | 1.64E+00 | 5.00E-04 |  |
| 1.00E+01 | 7.63E+11 | 1.85E+00 | 5.00E-04 |  |
| 1.00E+01 | 1.41E+08 | 3.68E+01 | 5.00E-04 |  |
| 1.00E+01 | 5.66E+08 | 1.16E+01 | 5.00E-04 |  |
| 1.00E+01 | 1.96E+09 | 3.41E+00 | 5.01E-04 |  |
| 1.00E+01 | 1.16E+11 | 2.18E+00 | 5.00E-04 |  |
| 1.00E+01 | 6.59E+11 | 1.95E+00 | 5.00E-04 |  |
| 1.00E+01 | 3.85E+12 | 4.08E+00 | 5.02E-04 |  |
| 1.00E+02 | 1.41E+08 | 6.57E+02 | 2.00E-03 |  |
| 1.00E+02 | 5.66E+08 | 1.51E+02 | 2.00E-03 |  |
| 1.00E+02 | 2.52E+09 | 3.68E+01 | 2.00E-03 |  |
| 1.00E+02 | 1.32E+11 | 2.45E+00 | 2.00E-03 |  |
| 1.00E+02 | 6.76E+11 | 4.03E+00 | 2.00E-03 |  |
| 1.00E+02 | 3.94E+12 | 8.37E+00 | 2.00E-03 |  |
| 1.00E+02 | 2.53E+13 | 9.76E+00 | 2.01E-03 |  |
| 1.00E+02 | 1.41E+08 | 4.90E+02 | 2.02E-03 |  |
| 1.00E+02 | 5.66E+08 | 1.51E+02 | 2.00E-03 |  |
| 1.00E+02 | 2.94E+09 | 3.78E+01 | 1.89E-03 |  |
| 1.00E+02 | 2.82E+11 | 4.53E+00 | 2.00E-03 |  |
| 1.00E+02 | 6.46E+11 | 4.61E+00 | 2.00E-03 |  |
| 1.00E+02 | 4.01E+12 | 1.03E+01 | 2.00E-03 |  |
| 1.00E+02 | 2.50E+13 | 1.16E+01 | 2.01E-03 |  |
| 1.00E+02 | 1.41E+08 | 6.57E+02 | 2.00E-03 |  |
| 1.00E+02 | 5.66E+08 | 1.51E+02 | 2.00E-03 |  |
| 1.00E+02 | 2.21E+09 | 3.67E+01 | 2.00E-03 |  |
| 1.00E+02 | 1.73E+11 | 3.67E+00 | 2.00E-03 |  |
| 1.00E+02 | 6.72E+11 | 4.74E+00 | 2.00E-03 |  |
| 1.00E+02 | 7.18E+11 | 4.90E+00 | 2.00E-03 |  |
| 1.00E+02 | 3.62E+12 | 8.21E+00 | 2.00E-03 |  |
| 1.00E+02 | 2.70E+13 | 1.14E+01 | 2.00E-03 |  |
| 1.00E+03 | 5.66E+08 | 1.71E+03 | 2.00E-03 |  |
| 1.00E+03 | 1.52E+10 | 6.29E+01 | 2.00E-03 |  |
| 1.00E+03 | 1.58E+11 | 8.12E+00 | 2.00E-03 |  |
| 1.00E+03 | 1.15E+12 | 7.43E+00 | 2.00E-03 |  |
| 1.00E+03 | 4.45E+12 | 1.25E+01 | 2.00E-03 |  |
| 1.00E+03 | 2.51E+13 | 2.09E+01 | 2.00E-03 |  |
| 1.00E+03 | 1.24E+14 | 4.33E+01 | 2.00E-03 |  |
| 1.00E+03 | 5.66E+08 | 1.71E+03 | 2.00E-03 |  |
| 1.00E+03 | 4.33E+09 | 3.87E+02 | 2.00E-03 |  |
| 1.00E+03 | 1.77E+10 | 6.57E+01 | 1.56E-03 |  |
| 1.00E+03 | 1.93E+11 | 9.51E+00 | 2.00E-03 |  |
| 1.00E+03 | 1.04E+12 | 6.90E+00 | 2.00E-03 |  |
| 1.00E+03 | 4.47E+12 | 1.05E+01 | 2.00E-03 |  |
| 1.00E+03 | 2.42E+13 | 1.91E+01 | 2.01E-03 |  |
| 1.00E+03 | 1.01E+14 | 4.53E+01 | 2.00E-03 |  |
| 1.00E+03 | 5.66E+08 | 1.71E+03 | 2.00E-03 |  |
| 1.00E+03 | 7.79E+09 | 3.81E+02 | 2.00E-03 |  |
| 1.00E+03 | 3.98E+10 | 4.25E+01 | 2.00E-03 |  |
| 1.00E+03 | 1.88E+11 | 8.53E+00 | 2.00E-03 |  |
| 1.00E+03 | 8.24E+11 | 8.00E+00 | 2.00E-03 |  |
| 1.00E+03 | 4.81E+12 | 1.23E+01 | 2.00E-03 |  |
| 1.00E+03 | 2.38E+13 | 1.48E+01 | 2.00E-03 |  |
| 1.00E+03 | 1.16E+14 | 3.20E+01 | 2.01E-03 |  |
| 1.00E+03 | 5.66E+08 | 1.71E+03 | 2.00E-03 |  |
| 1.00E+03 | 2.01E+09 | 4.53E+02 | 2.00E-03 |  |
| 1.00E+03 | 2.01E+09 | 4.53E+02 | 2.00E-03 |  |
| 1.00E+03 | 1.40E+10 | 6.16E+01 | 1.80E-03 |  |
| 1.00E+03 | 1.75E+11 | 8.37E+00 | 2.00E-03 |  |
| 1.00E+03 | 8.09E+11 | 8.53E+00 | 2.00E-03 |  |
| 1.00E+03 | 1.27E+12 | 8.74E+00 | 2.00E-03 |  |
| 1.00E+03 | 4.86E+12 | 1.22E+01 | 2.00E-03 |  |
| 1.00E+03 | 2.86E+13 | 2.49E+01 | 2.00E-03 |  |
| 1.00E+03 | 1.26E+14 | 3.92E+01 | 2.00E-03 |  |
| 1.00E+04 | 6.27E+09 | 1.54E+03 | 2.00E-03 |  |
| 1.00E+04 | 5.10E+10 | 1.66E+02 | 2.01E-03 |  |
| 1.00E+04 | 7.57E+10 | 1.40E+02 | 2.01E-03 |  |
| 1.00E+04 | 1.08E+11 | 1.02E+02 | 2.01E-03 |  |
| 1.00E+04 | 1.50E+12 | 1.94E+01 | 2.00E-03 |  |
| 1.00E+04 | 5.40E+12 | 1.80E+01 | 2.00E-03 |  |
| 1.00E+04 | 2.78E+13 | 2.56E+01 | 2.00E-03 |  |
| 1.00E+04 | 1.17E+14 | 3.36E+01 | 2.00E-03 |  |
| 1.00E+04 | 7.11E+14 | 8.45E+01 | 2.09E-03 |  |
| 1.00E+04 | 6.27E+09 | 1.54E+03 | 2.00E-03 |  |
| 1.00E+04 | 1.96E+10 | 5.84E+02 | 2.00E-03 |  |
| 1.00E+04 | 1.06E+11 | 1.05E+02 | 2.01E-03 |  |
| 1.00E+04 | 6.11E+11 | 3.20E+01 | 2.00E-03 |  |
| 1.00E+04 | 1.37E+12 | 2.00E+01 | 2.00E-03 |  |
| 1.00E+04 | 7.72E+12 | 2.54E+01 | 2.00E-03 |  |
| 1.00E+04 | 2.40E+13 | 1.99E+01 | 2.01E-03 |  |
| 1.00E+04 | 1.10E+14 | 4.86E+01 | 2.01E-03 |  |
| 1.00E+04 | 7.12E+14 | 9.02E+01 | 2.01E-03 |  |
| 1.00E+04 | 6.27E+09 | 1.54E+03 | 2.00E-03 |  |
| 1.00E+04 | 1.83E+10 | 6.37E+02 | 2.00E-03 |  |
| 1.00E+04 | 1.58E+11 | 1.00E+02 | 2.00E-03 |  |
| 1.00E+04 | 2.98E+11 | 5.51E+01 | 2.00E-03 |  |
| 1.00E+04 | 8.02E+11 | 2.14E+01 | 2.00E-03 |  |
| 1.00E+04 | 4.65E+12 | 1.67E+01 | 2.00E-03 |  |
| 1.00E+04 | 2.54E+13 | 2.16E+01 | 2.00E-03 |  |
| 1.00E+04 | 1.32E+14 | 4.37E+01 | 2.00E-03 |  |
| 1.00E+04 | 7.46E+14 | 7.10E+01 | 2.00E-03 |  |
| 1.00E+04 | 1.39E+10 | 7.68E+02 | 2.01E-03 |  |
| 1.00E+04 | 1.09E+11 | 1.02E+02 | 2.04E-03 |  |
| 1.00E+04 | 2.32E+11 | 8.45E+01 | 1.94E-03 |  |
| 1.00E+04 | 5.53E+11 | 3.67E+01 | 2.00E-03 |  |
| 1.00E+04 | 7.46E+11 | 2.71E+01 | 2.00E-03 |  |
| 1.00E+04 | 1.62E+12 | 1.74E+01 | 2.00E-03 |  |
| 1.00E+04 | 5.35E+12 | 2.01E+01 | 2.00E-03 |  |
| 1.00E+04 | 1.81E+13 | 2.45E+01 | 2.01E-03 |  |
| 1.00E+04 | 2.29E+13 | 3.11E+01 | 2.00E-03 |  |
| 1.00E+04 | 2.74E+13 | 3.11E+01 | 2.00E-03 |  |
| 1.00E+04 | 1.07E+14 | 4.29E+01 | 2.00E-03 |  |
| 1.00E+04 | 6.97E+14 | 8.57E+01 | 2.00E-03 |  |
| 1.00E+04 | 7.33E+14 | 7.14E+01 | 2.00E-03 |  |
| 1.00E+05 | 6.01E+10 | 1.77E+03 | 2.01E-03 |  |
| 1.00E+05 | 3.34E+11 | 8.78E+02 | 2.00E-03 |  |
| 1.00E+05 | 9.78E+11 | 1.34E+02 | 2.02E-03 |  |
| 1.00E+05 | 6.49E+12 | 5.55E+01 | 2.00E-03 |  |
| 1.00E+05 | 2.79E+13 | 4.12E+01 | 2.00E-03 |  |
| 1.00E+05 | 1.22E+14 | 5.31E+01 | 2.04E-03 |  |
| 1.00E+05 | 7.45E+14 | 8.65E+01 | 2.05E-03 |  |
| 1.00E+05 | 3.76E+15 | 1.31E+02 | 2.04E-03 |  |
| 1.00E+05 | 5.48E+15 | 1.10E+02 | 2.02E-03 |  |
| 1.00E+05 | 5.54E+10 | 1.98E+03 | 2.00E-03 |  |
| 1.00E+05 | 1.09E+11 | 1.01E+03 | 2.00E-03 |  |
| 1.00E+05 | 2.75E+11 | 4.25E+02 | 2.00E-03 |  |
| 1.00E+05 | 1.69E+12 | 1.00E+02 | 2.01E-03 |  |
| 1.00E+05 | 1.21E+13 | 5.47E+01 | 2.00E-03 |  |
| 1.00E+05 | 2.82E+13 | 4.98E+01 | 2.02E-03 |  |
| 1.00E+05 | 1.09E+14 | 5.35E+01 | 2.00E-03 |  |
| 1.00E+05 | 7.29E+14 | 9.43E+01 | 2.03E-03 |  |
| 1.00E+05 | 5.52E+15 | 9.35E+01 | 2.01E-03 |  |
| 1.00E+05 | 5.56E+10 | 1.65E+03 | 2.00E-03 |  |
| 1.00E+05 | 1.09E+11 | 9.59E+02 | 2.00E-03 |  |
| 1.00E+05 | 9.54E+11 | 1.47E+02 | 2.05E-03 |  |
| 1.00E+05 | 5.42E+12 | 5.39E+01 | 2.00E-03 |  |
| 1.00E+05 | 6.72E+12 | 5.27E+01 | 2.01E-03 |  |
| 1.00E+05 | 6.72E+12 | 5.27E+01 | 2.01E-03 |  |
| 1.00E+05 | 3.32E+13 | 4.86E+01 | 2.02E-03 |  |
| 1.00E+05 | 1.26E+14 | 5.35E+01 | 2.04E-03 |  |
| 1.00E+05 | 7.26E+14 | 1.11E+02 | 2.03E-03 |  |
| 1.00E+05 | 3.77E+15 | 1.36E+02 | 2.12E-03 |  |
| 1.00E+05 | 5.39E+15 | 1.47E+02 | 1.90E-03 |  |
| 1.00E+06 | 1.46E+12 | 9.92E+02 | 2.01E-03 |  |
| 1.00E+06 | 2.32E+12 | 5.76E+02 | 2.00E-03 |  |
| 1.00E+06 | 1.14E+13 | 2.34E+02 | 2.00E-03 |  |
| 1.00E+06 | 3.27E+13 | 1.55E+02 | 2.00E-03 |  |
| 1.00E+06 | 1.49E+14 | 1.14E+02 | 2.00E-03 |  |
| 1.00E+06 | 7.74E+14 | 1.27E+02 | 2.05E-03 |  |
| 1.00E+06 | 3.88E+15 | 1.64E+02 | 2.01E-03 |  |
| 1.00E+06 | 5.65E+15 | 9.68E+01 | 2.02E-03 |  |
| 1.00E+06 | 6.80E+11 | 1.65E+03 | 2.21E-03 |  |
| 1.00E+06 | 4.12E+12 | 4.29E+02 | 2.28E-03 |  |
| 1.00E+06 | 2.81E+13 | 1.65E+02 | 2.09E-03 |  |
| 1.00E+06 | 1.14E+14 | 1.00E+02 | 2.20E-03 |  |
| 1.00E+06 | 7.66E+14 | 8.82E+01 | 2.13E-03 |  |
| 1.00E+06 | 5.47E+15 | 9.88E+01 | 2.25E-03 |  |
| 1.00E+06 | 1.50E+12 | 9.35E+02 | 2.01E-03 |  |
| 1.00E+06 | 1.99E+12 | 6.45E+02 | 2.00E-03 |  |
| 1.00E+06 | 2.25E+12 | 6.45E+02 | 2.00E-03 |  |
| 1.00E+06 | 8.43E+12 | 2.56E+02 | 2.00E-03 |  |
| 1.00E+06 | 4.35E+13 | 1.60E+02 | 2.00E-03 |  |
| 1.00E+06 | 1.55E+14 | 1.26E+02 | 2.01E-03 |  |
| 1.00E+06 | 7.67E+14 | 1.36E+02 | 2.03E-03 |  |
| 1.00E+06 | 3.89E+15 | 1.31E+02 | 2.22E-03 |  |
| 1.00E+06 | 5.59E+15 | 1.09E+02 | 2.01E-03 |  |
| 1.00E+06 | 1.20E+16 | 1.38E+02 | 2.04E-03 |  |
|  | | | | |
| 2.50E+08 | 7.00E+14 | 4.57E+02 | 2.00E-03 | MD, Al |
| 2.50E+08 | 1.26E+15 | 2.49E+02 | 2.00E-03 |  |
| 2.50E+08 | 2.40E+15 | 2.34E+02 | 2.00E-03 |  |
| 2.50E+08 | 7.65E+15 | 1.58E+02 | 2.00E-03 |  |
| 2.50E+08 | 2.35E+16 | 2.93E+02 | 2.00E-03 |  |

**Supplementary References**

1 Livingston, J. D. The density and distribution of dislocations in deformed copper crystals. *Acta Metall.* **10**, 229-239 (1962).

2 Hildebrand, H. The effect of the initial dislocation density on dislocation multiplication and work-hardening characteristics of copper single crystals. *Phys. Status Solidi A* **12**, 239-249 (1972).

3 Van Drunen, G. & Saimoto, S. Deformation and recovery of [001] oriented copper crystals. *Acta Metall.* **19**, 213-221 (1971).

4 F. W. Young, J. On the Yield Stress of Copper Crystals. *J. Appl. Phys.* **33**, 963-969 (1962).

5 Yonenaga, I. & Sumino, K. Dislocation dynamics in the plastic deformation of silicon crystals I. Experiments. *physica status solidi (a)* **50**, 685-693 (1978).

6 Chiem, C. Y. & Duffy, J. Strain rate history effects in LiF single crystals during dynamic loading in shear. *Mater. Sci. Eng.* **48**, 207-222 (1981).

7 Chiem, C. Y. & Duffy, J. Strain rate history effects and observations of dislocation substructure in aluminum single crystals following dynamic deformation. *Mater. Sci. Eng.* **57**, 233-247 (1983).

8 Kumar, A., Hauser, F. E. & Dorn, J. E. Viscous drag on dislocations in aluminum at high strain rates. *Acta Metall.* **16**, 1189-1197 (1968).

9 Edington, J. W. The influence of strain rate on the mechanical properties and dislocation substructure in deformed copper single crystals. *The Philosophical Magazine: A Journal of Theoretical Experimental and Applied Physics* **19**, 1189-1206 (1969).

10 Meyers, M. A. *et al.* Laser-induced shock compression of monocrystalline copper: characterization and analysis. *Acta Mater.* **51**, 1211-1228 (2003).

11 Kattoura, M. & Shehadeh, M. A. On the ultra-high-strain rate shock deformation in copper single crystals: multiscale dislocation dynamics simulations. *Philosophical Magazine Letters* **94**, 415-423 (2014).

12 Wang, Z. Q., Beyerlein, I. J. & LeSar, R. Plastic anisotropy in fcc single crystals in high rate deformation. *Int. J. Plast.* **25**, 26-48 (2009).
